# The general nutrition practices of competitive powerlifters vary by competitive calibre and sex, weight, and age class

**DOI:** 10.1007/s00394-023-03233-6

**Published:** 2023-08-16

**Authors:** Andrew King, Kedric Kwan, Ivan Jukic, Caryn Zinn, Eric Helms

**Affiliations:** 1 Sport Performance Research Institute New Zealand (SPRINZ), Auckland University of Technology, 17 Antares Place, Mairangi Bay, Auckland, 0632 New Zealand; 2 School of Engineering, Computer and Mathematical Sciences, Auckland University of Technology, Auckland, New Zealand; 3 Department of Exercise Science and Health Promotion, Muscle Physiology Laboratory, Florida Atlantic University, Boca Raton, FL, USA

**Keywords:** Powerlifting, Nutrition practices, IIFYM, Resistance training, Athletes

## Abstract

**Purpose:**

To characterise self-reported nutrition practices and beliefs of powerlifters.

**Methods:**

Actively competing male (n = 240) and female (n = 65) powerlifters completed a cross-sectional online survey of self-reported nutrition practices across the competitive cycle, within specific competitive phases, and hard and easy training days. Data are presented as number (*n*) and percentage (%) of all powerlifters practicing a given strategy followed by a % of responses reporting various practices or beliefs within this strategy. Differences in categorical sub-groups (sex, age, and weight class; and competitive calibre) were analysed with a chi-square test and denoted where significant (p ≤ 0.05).

**Results:**

Most powerlifters reported following a specific diet long-term (n = 203, 66.6%) of which If It Fits Your Macros (IIFYM)/flexible dieting was most common (n = 159, 78.3%). Over half reported introducing a special diet for a competitive phase (n = 162, 53.1%), of which IIFYM/flexible dieting was most followed for competition preparation (n = 80, 63%) and off-season (n = 48, 71.6%). Compared to normal dietary intake, most reported eating more on harder training days (n = 219, 71.8%) and refraining from eating less on easier training days (n = 186, 61%).

**Conclusions:**

IIFYM/flexible dieting is commonly followed by powerlifters to support performance and body composition goals. Females seemed to report more often restricting energy and dieting for body composition reasons than males. Powerlifters tailor their energy intake on harder training days to the higher training demands but refrain from reducing energy intake on rest/easier training days.

**Supplementary Information:**

The online version contains supplementary material available at 10.1007/s00394-023-03233-6.

## Introduction

Powerlifting is a strength sport consisting of three lifts: the back squat, bench press, and deadlift. Powerlifters are delineated by competitive federation (e.g., drug-tested, or not), the use of supportive lifting equipment or not (i.e., equipped, or classic), and their age, sex, and weight category. Performance is determined by the cumulative total weight lifted from the three lifts; therefore, powerlifters manipulate their training and nutrition with the intention of enhancing strength. Resistance training, as completed by powerlifters, can lead to fibre type transition, neuromuscular adaptation (e.g., increased motor unit recruitment), and increases in fat free mass (FFM) [1]. Skeletal muscle hypertrophy, specifically, is a strong predictor of powerlifting performance [2]. Consequently, dietary strategies for powerlifters are of acute (e.g., before, during, and after a training session) and longitudinal (e.g., across one or more competitive phases) interest. For instance, acute dietary strategies are of interest to fuel and recover from individual training bouts (e.g., aid performance and recovery). Additionally, powerlifters in weight classes with an upper weight limit may also use acute nutrition strategies to induce a rapid weight cut in the day/s before competition. On the other hand, chronic dietary strategies are of interest to powerlifters to aid in the optimisation of body composition (i.e., body weight manipulation such as periods of intentional gain/loss and lean mass accumulation).

One such nutritional strategy is the concept of nutritional periodisation, which can be summarised as the planned, purposeful, and strategic use of nutritional interventions to enhance adaptations across a training session or plan, or to enhance long term performance [3]. Nutritional periodisation is thoroughly studied in the context of endurance exercise [3], but nutritional periodisation for strength athletes is less researched [4]. Different powerlifting competitive phases, such as the off-season/general preparation and competition preparation phases, have distinct theoretical considerations [5]. Off-season and general preparation phases typically consist of higher training volumes (i.e., the total amount of mechanical work performed); therefore, higher (absolute or relative to body mass) energy intakes (EI) are required to match the demands of the increased workload. Additionally, a small calorie surplus is advised to maximise lean mass gains, emphasising the need for adequate EI during the off-season/general preparation phase [6]. Conversely, competition preparation in the weeks and months before competition usually culminates in a training volume taper with more lifts completed at a higher percentage of 1-repetition maximum closer to competition [7]. Thus, EI needs may be lower during the competition preparation phase to match lower energetic demands of training. Powerlifters commonly induce gradual weight loss via caloric restriction in the weeks/months preceding competition [8, 9]. In addition, powerlifters may combine gradual dieting with rapid weight loss strategies in the days/hours preceding weigh-in on competition day to compete in a weight class lower than their habitual body weight would allow [8, 9]. Overall, there are several competitive phases in powerlifting that require different nutrition strategies depending on training volume performed and body composition goals of the athlete.

Current resistance training nutrition recommendations are to ingest 4–7 g/kg bodyweight of carbohydrate (CHO) and 1.6–2.2 g/kg bodyweight protein per day [10, 11]. Dietary fats are less emphasised, but guidelines advise the remaining 20–30% of daily caloric intake be allotted to them, sourced from mono- and poly-unsaturated fats [12]. These guidelines exist because sufficient dietary protein is necessary to optimise muscle mass accretion [11] and adequate dietary CHO intake can in some circumstances enhance aspects of resistance training performance [13]. The If It Fits Your Macros (IIFYM) diet is common within various fitness communities and is characterised as an eating approach designed to reach specific daily targets in grams of protein, CHO, and fats without a restriction on food source [14, 15]. In addition, IIFYM is often paired with ‘flexible’ dieting, which is an approach to dieting that represents a more moderate, balanced approach to dieting for weight loss, and is generally contrasted by ‘rigid’ dieting where the dieter is either ‘on’ or ‘off’ a diet [16]. However, it is not known whether these dietary approaches assist powerlifters with meeting current sport nutrition guidelines, or if they implement unique nutritional periodisation approaches. One study quantified nutrition intakes of powerlifters in the off-season [17]; however, no study has investigated the nutrition strategies of powerlifters during the competitive season. Given their competitive phase-specific training demands and goals (i.e., maximal strength in the three competition lifts), the aim of this study was to survey the nutrition practices of competitive powerlifters around the competitive cycle, and individual training sessions (harder versus easier/rest days). This exploration allows for an account of current practices used by powerlifters, their underlying rationale, and a discussion of how practices relate to current sport nutrition guidelines.

## Methods

An open invite, anonymous international survey was developed to investigate the nutrition practices and beliefs of competitive powerlifters. Data collection for this study was completed between November 2020 and February 2021. The methods of this survey are reported in accordance with the Checklist for Reporting Results of Internet E-surveys (CHERRIES) [18]. The study protocol was approved by the Auckland University of Technology Ethics Committee (20/312).

### Survey development and design

The structure and content of the questionnaire was based on previously published work investigating the nutrition practices of elite race walkers [19], with content either adapted or added to suit a powerlifting context. A first draft of the survey was piloted with a convenience sample of powerlifters (n = 8) who provided feedback. Based on feedback from pilot testing, the survey was modified to improve content and readability. The final version of the survey was built and distributed online using Qualtrics software (Seattle, WA, USA) and contained 81 questions in total. Display logic and exclusive answers were used to build a custom path through the questionnaire dependent on the participant’s answer such that 30–40 questions over 16–22 pages were answered by each participant. Participants were shown 1–3 questions per page and a back button was enabled that allowed participants to amend/change previous answers.

The questionnaire was split into ten sections, of which the first five (i.e., general nutrition practices) are of interest in this manuscript. In order of appearance, Sect. 1 covered participants’ descriptive characteristics (e.g., nation of competition and competitive division) and training history (e.g., powerlifting experience and competitive calibre). Section 2 covered general dietary themes for the overall competitive cycle. Section 3 covered the general dietary themes for specific competitive phases (e.g., off-season, competition preparation). Sections 4 and 5 covered harder and easier training day nutrition practices, respectively. A full transcript of the questionnaire with display logic can be found in Supplementary File I.

Definitions for concepts were provided to participants in the survey. A hard training day was defined as a high volume and/or high intensity session. A rest/easier training day was defined as passive or active recovery or lower volume accessory days where none of the three powerlifting lifts are completed. A figure (Page 32 of Supplementary File I) was presented to participants visualising a generic, periodised competition cycle and competitive phases within the cycle (i.e., off-season, competition preparation, competition, and transition) [5]. The competition preparation phase was presented as including specific preparation for, and then a taper into, competition. The competition phase was defined as including the day of competition and the 48 h preceding. The transition phase was defined as occurring immediately post-competition, which led into an off-season or general preparation. The off-season was presented as including general preparation for competition, which preceded a competition preparation phase and followed a transition phase.

### Survey distribution and sample selection

The survey was distributed via advertisement on social media with an accompanying internet link to the anonymous survey. A downloadable information sheet detailing the purpose, content, and length of the survey; how, when, and where data will be stored; and who the investigators were was displayed as the first page before commencing any survey questions. Due to the survey being anonymous, participants were advised in the information sheet that participation was voluntary, and consent was provided by submitting the completed survey. Participant inclusion criteria were to (a) be 18 years of age or older (which excluded sub-junior age class powerlifters less than 18 years of age from participating in the present study), and (b) have competed in a drug-free sanctioned powerlifting competition within the previous 18 months.

### Statistical analysis

Only fully completed questionnaires were analysed. Missing data checks were performed to verify data integrity. Descriptive data were presented as number (*n*) and percentages (*%*). Categorical data were assessed by chi-square test and Cramer’s V (φ_*c*_). Where > 20% of cells had an expected count of less than 5, Fisher’s exact test was used. For the weight-class subgroup (4 × 2 contingency table), a follow-up individual chi-square test (or Fisher’s exact test where the > 20% expected count rule was violated) with Holm-Bonferroni correction were performed when a statistically significant result was observed. Statistical significance was set at p ≤ 0.05. Data were prepared and analysed in SPSS (version 27.0; IBM Corp, Armonk, NY), and follow-up chi-square and Fisher’s tests were completed in R language for statistical computing (R Foundation for Statistical Computing, Vienna, Austria, 2021) using the “*Fifer2*” package (https://github.com/dustinfife/fifer2/).

Participants could select multiple answers for most questions; therefore, the percentage of responses for some questions could add up to more than 100%. Text answers to “other” responses were grouped into common themes/responses by the primary investigator (AK). Responses were analysed by sub-groups based on competitive division (males vs. females), age class (sub-juniors and juniors [SJ + J] vs. open and masters [O + M]), weight class (women’s under 63, 57, 52, and 47 kg classes [W63-] vs. women’s under 72, 84 kg classes and 84 kg plus class [W72 +] vs. men’s under 83, 74, 66, 59, and 43 kg classes [M83-] vs. men’s under 93, 105, 120 kg classes and 120 kg plus class [M93 +]), and competitive calibre (i.e., IPF points where higher values indicate stronger powerlifters relative to bodyweight) from the best 3-lift total in competition (less than 80 IPF points [79- IPF] vs. 80 IPF points or more [80 + IPF]).

## Results

**Fig. 1:**
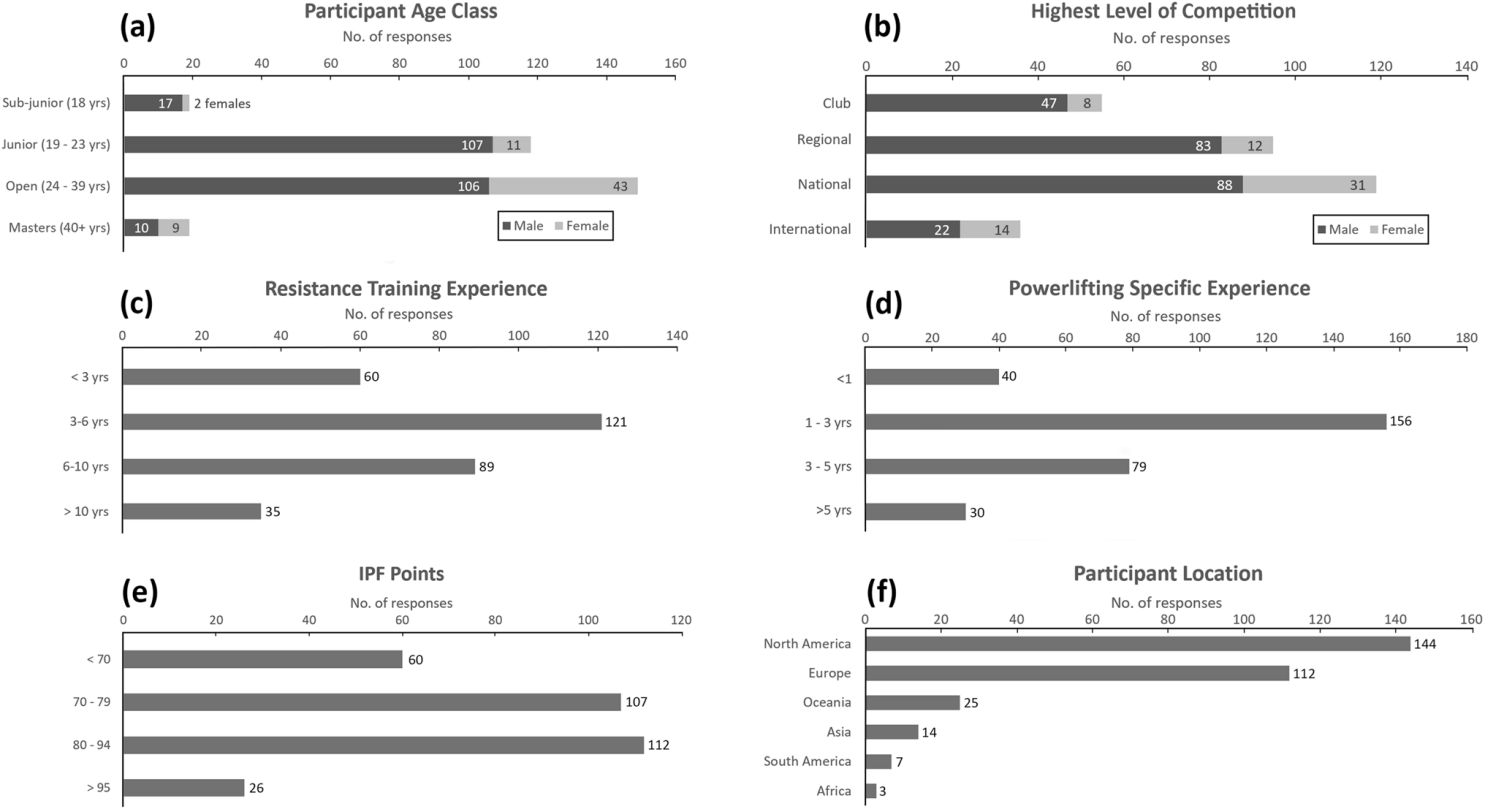
Descriptive participant characteristics of **a** age class, **b** competitive calibre, **c** resistance training experience in years, **d**, powerlifting specific experience in years, **e** International Powerlifting Federation (IPF) points based on best total in competition, and **f** participants location. For (**f**), participants were from 47 countries, which were grouped by continent

There were 385 responses, of which 305 (240 male and 65 female) fully completed the survey (79.8% completion rate) and were included in the analysis. Most participants resided in the United States (n = 115, 37.7%), Canada (n = 29, 9.5%), United Kingdom (n = 22, 7.2%), and New Zealand (n = 19, 6.2%), while the rest were from a variety of other countries (n = 120, 39.3%). Most participants competed in the open age class (n = 149, 48.9%), followed by juniors (n = 118, 38.7%), sub-junior (n = 19, 6.2%), and masters (n = 19, 6.2%). Descriptive characteristics are presented in Fig. 1.

### Question 1: nutrition for the competitive cycle

**Fig. 2:**
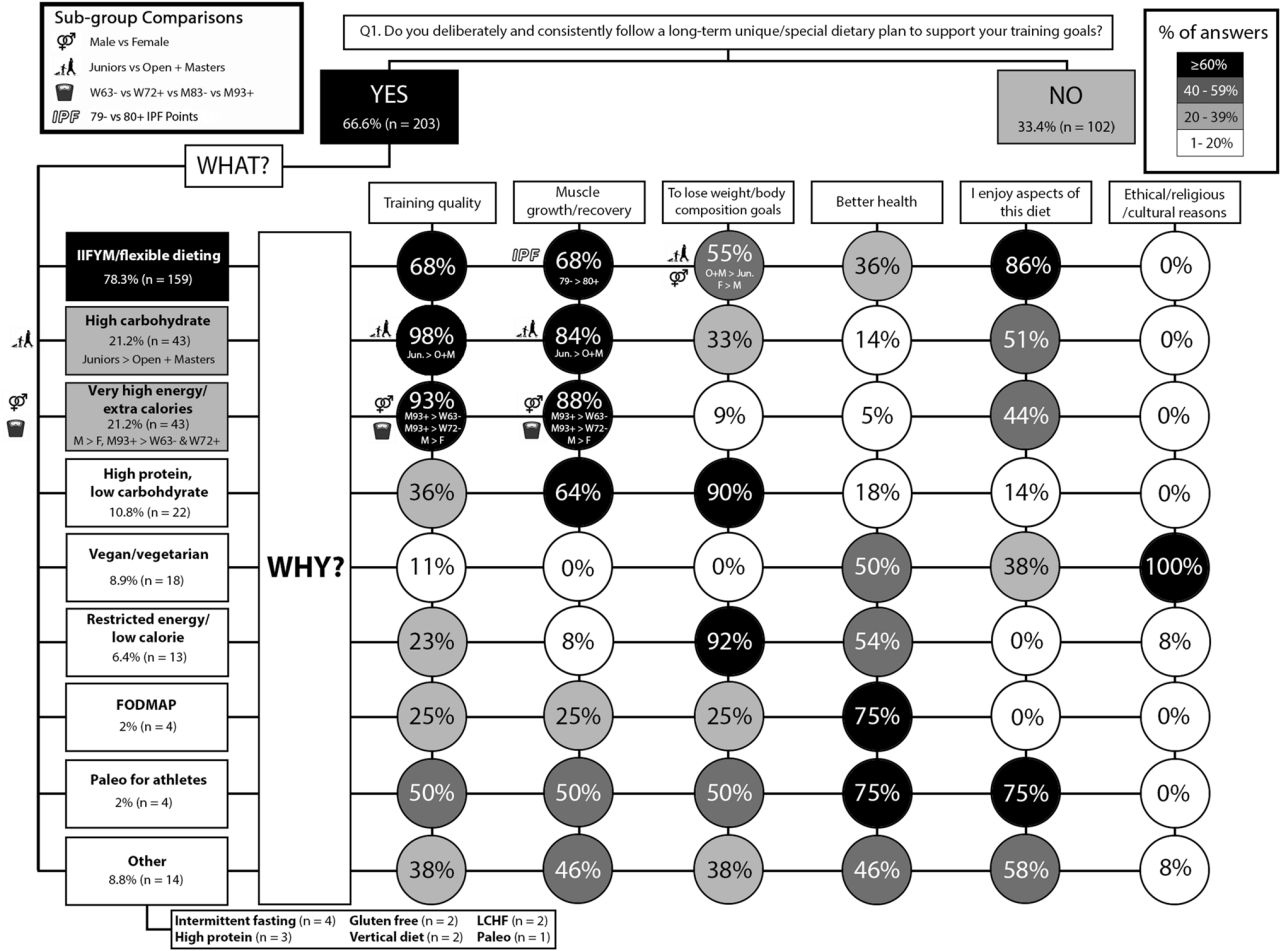
Q1: Overall dietary practices across the year. The prevalence of a specific and consistent overall long-term dietary approach (e.g., high carbohydrate) across the year and the reasons for following them in 305 actively competing powerlifters. Percentages (%) are presented as the proportion of all participants that chose a specific answer (YES/NO), followed by a % of responses reporting various practices and reasons within these strategies. Number (n) of participants has been provided. Answer boxes and circles are colour coded based on the % of responses: ≥ 60%, black box with white font; 40–59%, dark grey box with white font; 20–39%, light grey with black font; < 20%, white box with black font. Symbols are used to indicate statistical significance between sub-groups for sex (vector sex symbol), age class (human life cycle symbol), weight class (weight scale symbol), and International Powerlifting Federation (IPF) points (IPF symbol). Where significant differences were detected, the direction of difference is indicated in the corresponding box/circle (e.g., males reported more often than females would be indicated by M > F). *IIFYM* If It Fits Your Macros, *LCHF* low carbohydrate, high fat

Overall, 66.6% (n = 203) of participants reported following a long-term special/unique dietary plan (Fig. 2). Of these, 78.3% (n = 159) reported following an IIFYM/flexible dieting approach, followed by a high CHO (21.2%, n = 43) and a high energy approach (21.2%, n = 43). SJ + J reported following a long-term high CHO diet more than O + M (p = 0.027; φ_*c*_ = 0.127) for training quality (p = 0.040; φ_*c*_ = 0.117) and muscle growth/recovery (p = 0.038; φ_*c*_ = 0.119). Males more often reported following a high energy diet than females (p = 0.001; φ_*c*_ = 0.188) for training quality (p = 0.002; φ_*c*_ = 0.178) and muscle gain/recovery (p = 0.003; φ_*c*_ = 0.172). M93 + more often reported following a high energy diet than W63- (p = 0.049; φ_*c*_ = 0.209) and W72 + (p = 0.014; φ_*c*_ = 0.230). M93 + more often reported following a high energy diet for training quality (p = 0.048, φ_*c*_ = 0.182; p = 0.027, φ_*c*_ = 0.203) and muscle growth/recovery (p = 0.048, φ_*c*_ = 0.182; p = 0.027, φ_*c*_ = 0.203) than W63- and W72 + , respectively. Females more often than males (p = 0.045; φ_*c*_ = 0.115) and 79- IPF more often than 80 + IPF (p = 0.050; φ_*c*_ = 0.112), reported following an IIFYM/flexible dieting approach for muscle growth/recovery. O + M more often reported following an IIFYM/flexible diet for weight/body composition goals than SJ + J (p = 0.049; φ_*c*_ = 0.218). The results for nutrition practices across the competitive cycle are presented in Fig. 2. The information sources used to inform nutrition practices across the competitive cycle are presented in Table 1.

**Table 1:** Source of information informing nutrition practices of powerlifters

Question	Answer	Source of Information (*n*)								
		No specific source	I read/watched it	Coach	Sport Nutr	Dietician	Scientist	Friend	Training Partner	Other
Competitive Cycle: Do you follow a long term special/unique dietary plan?	Yes (n = 203)	162	21	9	6	4	6	9	5	4
Do you follow a unique/special dietary plan during competition preparation?	Yes (n = 127)	122	3	1	2	2	1	0	0	0
Do you follow a unique/special dietary plan during competition?	Yes (n = 109)	103	4	3	4	1	2	0	0	0
Do you follow a unique/special dietary plan during off-season?	Yes (n = 67)	65	1	0	1	1	1	0	0	0
Do you intentionally eat more food/calories on all harder training days?	Yes (n = 219)	61	101	73 †	47	15 ‡	21	21	22	2
	No (n = 86)	52	20	16	3	6	6	2	3	9
Do you intentionally eat less food/calories on rest/easier training days?	Yes (n = 119)	37	43	30	28	10	15	6	9	10
	No (n = 186)	97	60 ║§	36	11	7	6	5	7	9

*Sport Nutr.* Sport Nutritionist

“*Other*” includes medical doctor, physiotherapist, family member, and personal trainer

Significant differences (p ≤ 0.05) marked as: † females > males; ║males > females; ‡ 80 + IPF Points > 79- IPF Points; § M93 +  > W72 +

### Question 2: nutrition for specific competitive phases

#### Competition preparation

**Fig. 3:**
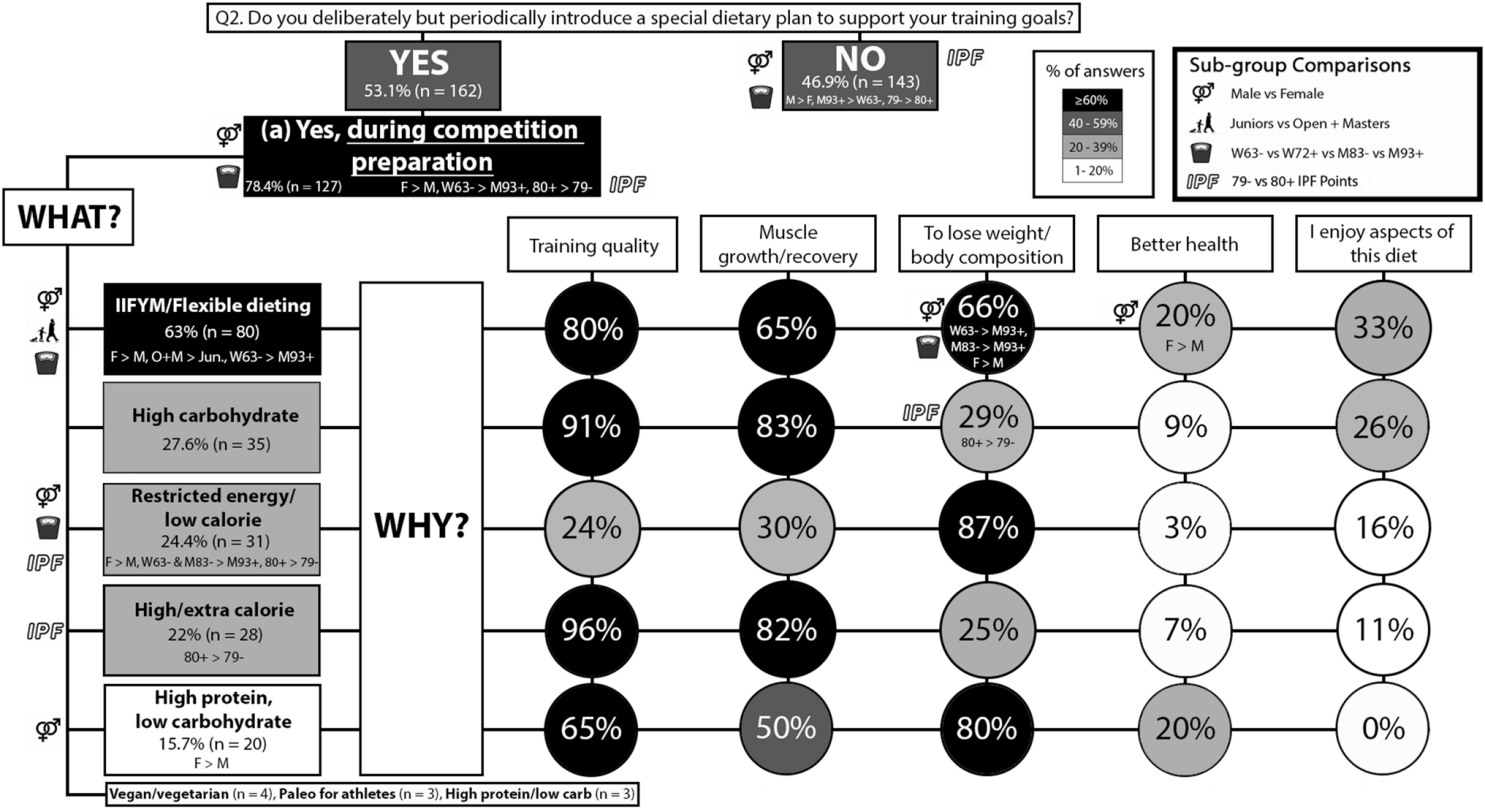
Q2 **a**: Dietary practices for the competition preparation phase. The prevalence of introducing a unique dietary approach for a specific competition phase (YES/NO) followed by the competition preparation phase and the reasons for following them. *IIFYM* If It Fits Your Macros. Description of how to interpret is in the Fig. 2 caption

Overall, 46.9% (n = 143) reported no introduction of a special/unique dietary plan during for a specific competition phase, which was reported more often by males than females (p = 0.018; φ_*c*_ = 0.136), M93 + more often than W63- (p = 0.016; φ_*c*_ = 0.185), and 79- IPF more often than 80 + IPF (p = 0.003; φ_*c*_ = 0.168). Conversely, 53.1% (n = 162) of powerlifters followed a special/unique dietary plan in one or more specific competitive phase/s. A special/unique diet during competition preparation was reported by 78.4% (n = 127) of powerlifters. Females more often reported than males (p = 0.002; φ_*c*_ = 0.178); W63- more often than M93 + (p = 0.001; φ_*c*_ = 0.329) and M83- (p = 0.016; φ_*c*_ = 0.251); and 80 + IPF more often than 79- IPF (p = 0.001; φ_*c*_ = 0.194), reported a special/unique dietary plan during competition preparation. During competition preparation, females more often than males (p = 0.002; φ_*c*_ = 0.181), W63- more often than M93 + (p = 0.002; φ_*c*_ = 0.312), and O + M more often than SJ + J (p = 0.038; φ_*c*_ = 0.119), reported following an IIFYM/flexible diet. Females more often reported a restricted energy (p = 0.042; φ_*c*_ = 0.116) and a high protein, low CHO (p = 0.001; φ_*c*_ = 0.186) diet than males. W63- (p = 0.018; φ_*c*_ = 0.270) and M83- (p = 0.044; φ_*c*_ = 0.178) more often reported a restricted energy diet than M93 + . 80 + IPF more often reported a restricted energy (p = 0.023; φ_*c*_ = 0.130) and high energy (p = 0.012; φ_*c*_ = 0.144) diet than 79- IPF. Regarding the reason for a specific diet, females more often reported an IIFYM/flexible diet for the purpose of losing weight/body composition goals (p = 0.002; φ_*c*_ = 0.342) and better health (p = 0.007; φ_*c*_ = 0.304) than males. W63- (p = 0.001; φ_*c*_ = 0.641) and M83- (p = 0.010; φ_*c*_ = 0.426) more often reported following an IIFYM/flexible diet for the purpose of losing weight/body composition goals than M93 + . 80 + IPF more often reported following a high CHO diet to lose weight/body composition goals than 79- IPF (p = 0.028; φ_*c*_ = 0.334). The results for competition preparation are presented in Fig. 3.

### Competition

**Fig. 4:**
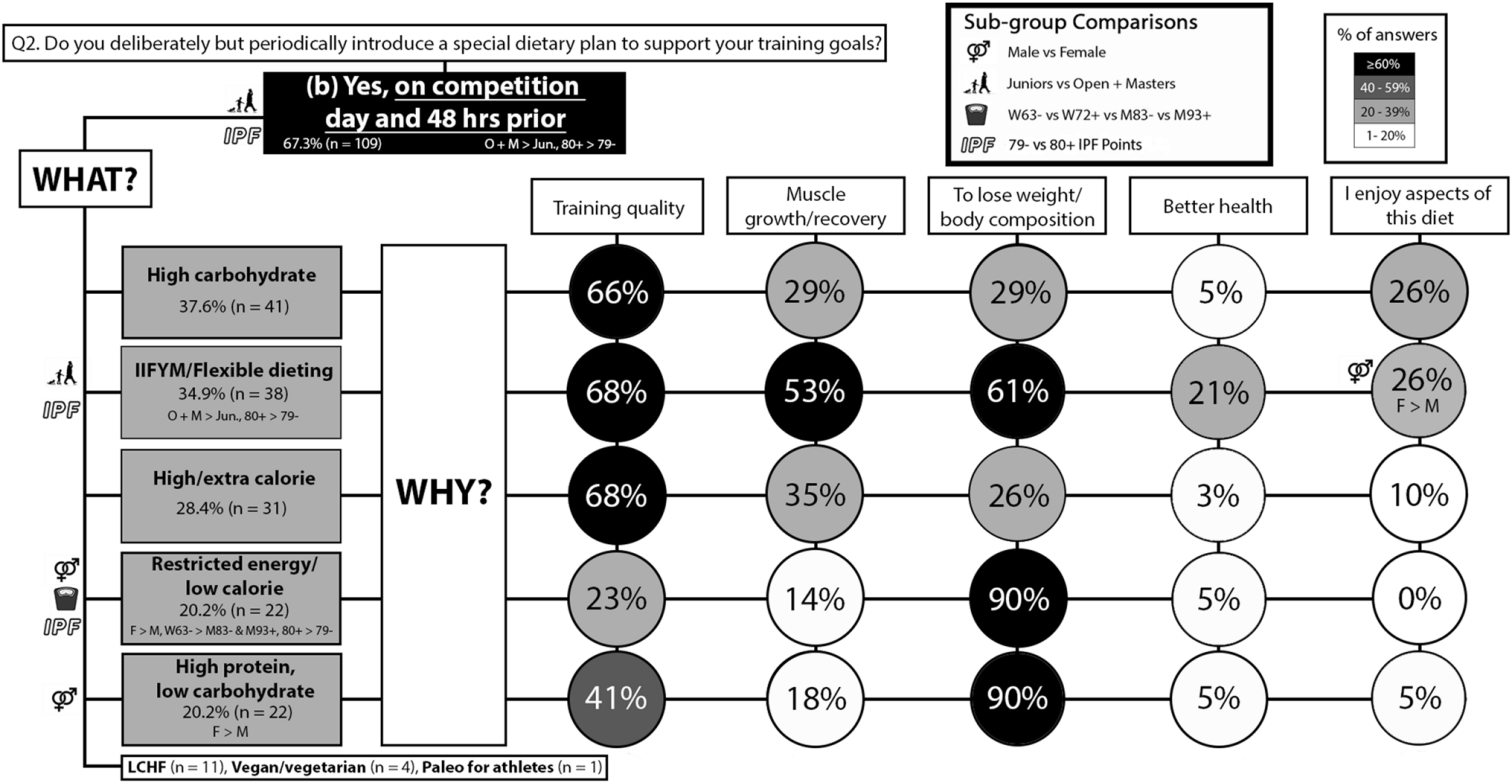
Q2 **b**: Dietary practices for the competition phase (day of competition and the 48 h preceding). The prevalence of introducing a unique dietary approach for the competition phase and the reasons for following them. Please note that the initial YES/NO responses are reported in Fig. 3 only. *IIFYM* If It Fits Your Macros. Description of how to interpret is in the Fig. 2 caption

During competition, O + M more often than SJ + J (p = 0.017; φ_*c*_ = 0.137) and 80 + IPF more often than 79- IPF (p = 0.001; φ_*c*_ = 0.202), reported the introduction of a special/unique dietary plan. O + M more often than SJ + J reported following an IIFYM/flexible diet (p = 0.001; φ_*c*_ = 0.251). 80 + IPF more often than 79- IPF reported following IIFYM/flexible dieting (p = 0.043; φ_*c*_ = 0.116) and restricted energy (p = 0.025; φ_*c*_ = 0.128). Females more often than males reported restricted energy (p = 0.020; φ_*c*_ = 0.133) and high protein, low carbohydrate (p = 0.025; φ_*c*_ = 0.128) diet. W63- more often than M83- (p = 0.011; φ_*c*_ = 0.140) and M93 + (p = 0.010; φ_*c*_ = 0.155) reported restricted energy. Regarding the reason for following a specific diet, females more often than males reported following an IIFYM/flexible diet for enjoyment reasons (p = 0.012; φ_*c*_ = 0.419). The results for the competition phase are presented in Fig. 4.

### Off-season

**Fig. 5:**
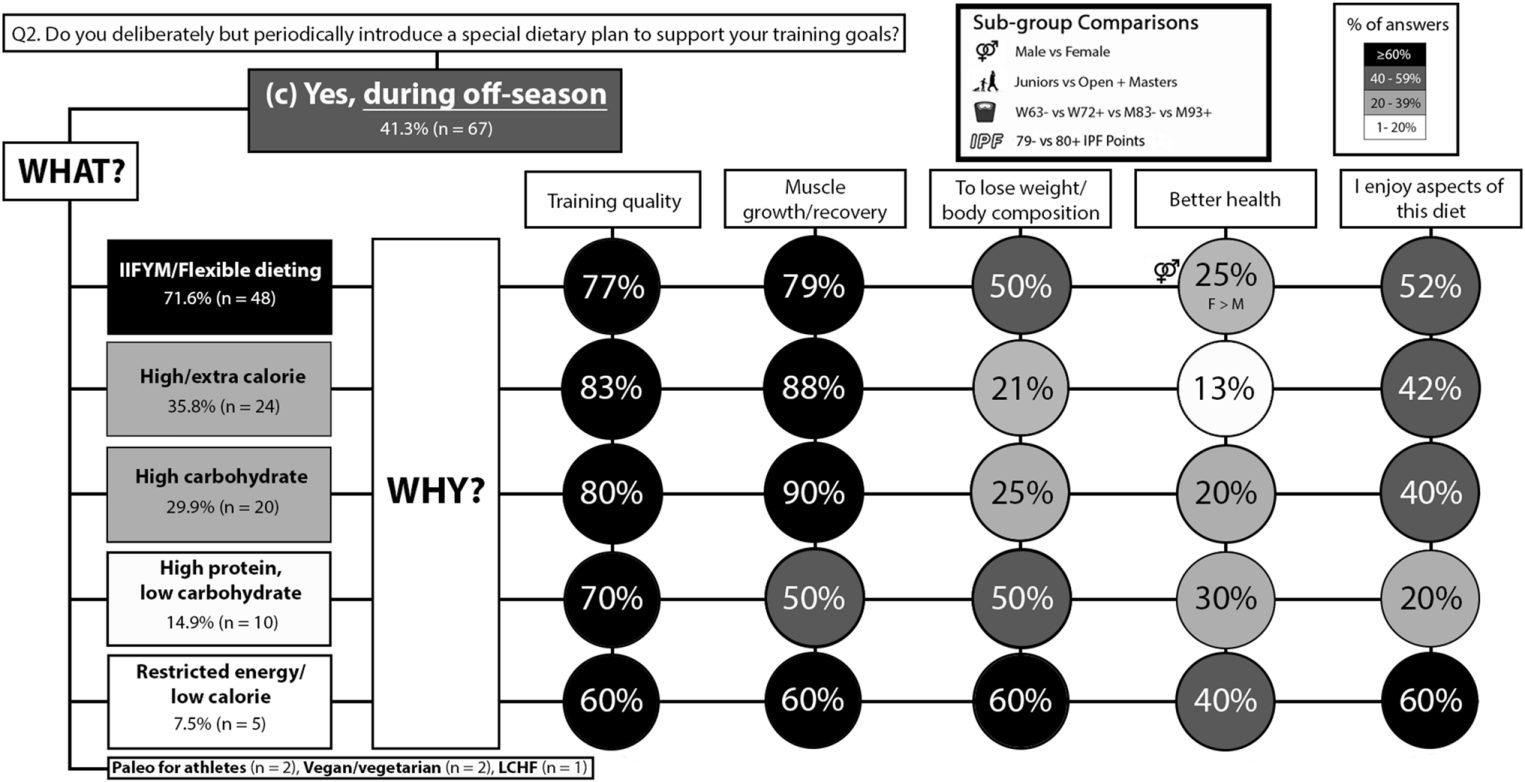
Q2 **c**: Dietary practices for the off-season phase. The prevalence of introducing a unique dietary approach for the off-season and the reasons for following them. Please note that the initial YES/NO responses are reported in Fig. 3 only. *IIFYM* If It Fits Your Macros, *LCHF* low carbohydrate, high fat. Description of how to interpret is in the Fig. 2 caption

During the off-season, females more often reported following an IIFYM/flexible diet for the purpose of bettering their health than males (p = 0.001; φ_*c*_ = 0.476). The results for the off-season phase are presented in Fig. 5. The information sources used to inform nutrition practices for specific competitive phases are presented in Table 1. The results for transition and return from injury are reported in Supplementary File II.

### Question 3: harder training days

**Fig. 6:**
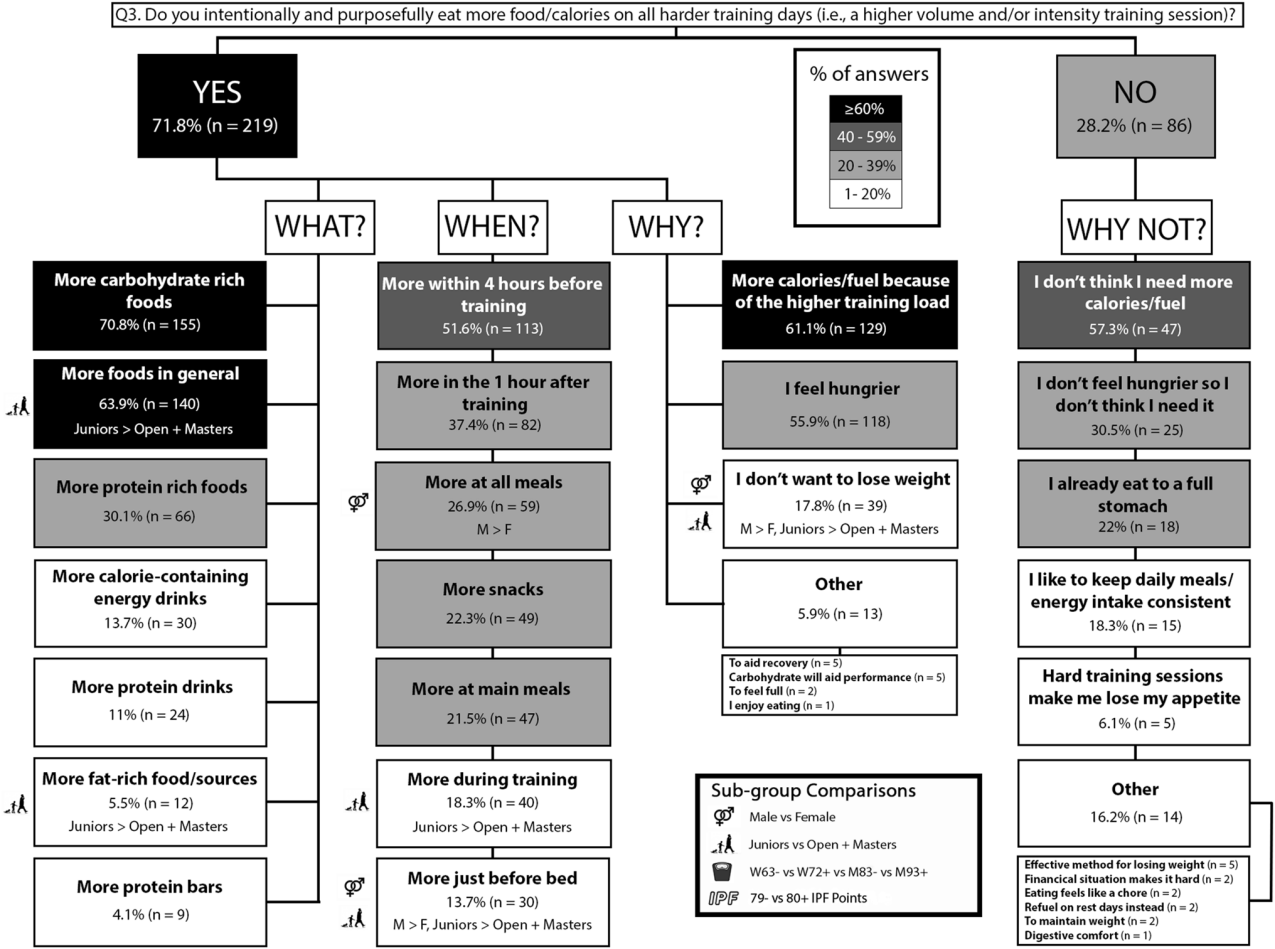
Q3: Nutrition on hard training days. The prevalence of specific nutrition practices on harder (i.e., higher volume/intensity) training days and the reasons for following them. Description of how to interpret is in the Fig. 2 caption

Overall, 71.8% (n = 219) of participants reported eating more food on harder training days via eating more CHO-containing foods (n = 155) or more foods in general (n = 150), and most participants reported eating more foods to get in the energy required to support the higher training volume (n = 129). SJ + J more often reported eating more foods in general (p = 0.019; φ_*c*_ = 0.134) and more fat rich foods/sources (p = 0.033; φ_*c*_ = 0.122) than O + M. Males more often reported eating more at all meals (p = 0.020; φ_*c*_ = 0.133) and just before bed (p = 0.039; φ_*c*_ = 0.118) than females. SJ + J more often reported eating more during training (p = 0.040; φ_*c*_ = 0.118) and before bed (p = 0.012; φ_*c*_ = 0.144), than O + M. Males more often than females (p = 0.026; φ_*c*_ = 0.126) and SJ + J more often than O + M (p = 0.010; φ_*c*_ = 0.148), reported eating more to avoid weight loss. The results for nutrition practices on harder training days are presented in Fig. 6.

Females more often than males informed the practice of eating more on harder training days with information from their coach (p = 0.035; φ_*c*_ = 0.121). 80 + IPF more often than 79- IPF informed their decision to eat more on harder training days with information from a dietician (p = 0.025; φ_*c*_ = 0.128). The information sources used to inform harder training day nutrition practices are presented in Table 1.

### Question 4: easier training days

**Fig. 7:**
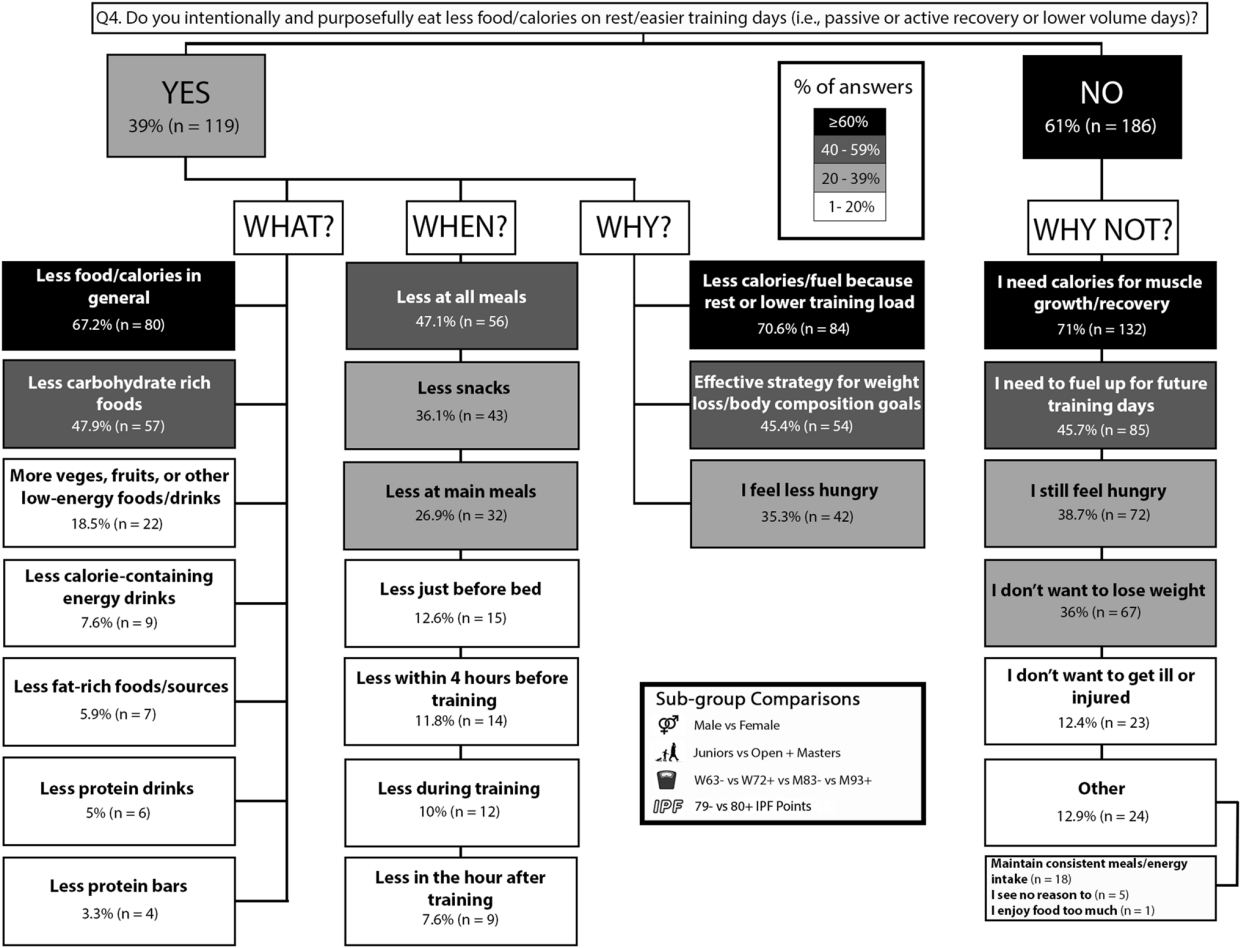
Q4: Nutrition on rest/easier training days. The prevalence of specific nutrition practices on rest/easier training days and the reasons for following them. Description of how to interpret is in the Fig. 2 caption

Overall, 61% (n = 186) of participants did not eat less food on easier training days. The most common reason was that calories were needed to support muscle growth/recovery (71%, n = 132). Of the 39% (n = 119) that did reduce food intake on easier training days, 67.2% (n = 80) reported eating less at all meals and 70.6% (n = 84) ate less because of the lower training load. There were no significant sub-group differences for easier training day nutrition practices (p > 0.05). The results for easier training day nutrition practices are displayed in Fig. 7.

Males more often than females (p = 0.017; φ_*c*_ = 0.137) and M93 + more often than W72 + (p = 0.046; φ_*c*_ = 0.162), reported informing their decision not to eat less on easier training days based on information they had read or watched. The information sources used to inform easy training day nutrition are presented in Table 1.

## Discussion

The purpose of the current study was to explore the general nutrition practices in competitive powerlifters. To our knowledge, this is the first study to characterise the general nutrition practices of powerlifters across competitive calibre, and sex, weight and, age class. There are several key findings: (1) Powerlifters use IIFYM/flexible dieting year-round, and during the competition preparation and off-season phases for performance and diet enjoyment; (2) nutrition strategies differ between competitive phases and there are various approaches to the competition phase; (3) female powerlifters are more conscious of EI than male counterparts; and (4) powerlifters focus on adequate EI on hard training days, but refrain from tailoring EI to the lower training workload on rest/easier training days.

### Nutrition practices and the competitive cycle

IIFYM is popular in various fitness communities and the findings of the present study indicate that this approach is also popular with powerlifters as a long-term approach to the diet (Fig. 2), and within the competition preparation (Fig. 3) and off-season (Fig. 5) phases. IIFYM is characterised as a meal plan constructed to reach specific daily targets in grams of protein, CHO, and fats without a restriction on food source [14, 15]. Given this, IIFYM allows for more dietary inclusion and variety, and supports periods of weight gain, loss, or maintenance [14]. In the current study, IIFYM was paired with flexible dieting, the latter of which can be considered a distinct concept specific to weight loss. Cognitive restraint during phases of weight loss, to create a caloric deficit, is required on behalf of the dieter, and the approach to cognitive restraint has been characterised as an individual exerting more ‘flexible’ or more ‘rigid’ control, as scored on the three-factor eating questionnaire [20]. A rigid approach to dieting describes an all or nothing approach to eating behaviours and dieting, where ‘off-limit’ foods are avoided or eliminated and ‘diet’ foods are emphasised, narrowing the variability of foods consumed [21]. Flexible control represents a more moderate, balanced approach to dieting for weight loss that includes eating behaviours such as taking smaller servings of food than desired to regulate weight and including a variety of foods in limited quantities [16]. Thus, flexible dieting for weight loss can be considered more consistent with, but conceptually different to, the IIFYM approach to organising the diet.

IIFYM emphasises reaching daily targets for macronutrients but does not necessarily consider micronutrient intakes and it is presently unknown whether powerlifters reach the recommended dietary allowance (RDA) for micronutrients with an IIFYM approach. Micronutrient intakes below the RDA have been reported in bodybuilders [22] but micronutrient intakes have not been quantified in powerlifters. Recently, in a cohort of bodybuilders, Ismaeel et al., [14] reported intakes of vitamins A, D, and E; potassium; and fibre below the RDA, with similar prevalence between males following a macronutrient-based diet and those following a more rigid, ‘strict’ diet. Additionally, while there was a low sample size of female bodybuilders, those following an IIFYM approach reported higher, but still inadequate, micronutrient values compared to strict dieting females, and all females reported iron intakes below the RDA [14]. The prevalence of iron deficiency is 15–35% in athletic, female populations [23]. Female athletes consuming lower EIs, practicing vegan or vegetarian diets, and participating in endurance sports are notable factors in the prevalence of iron deficiency [24]; however, it is presently unknown to what degree female powerlifting athletes are affected. Future research is necessary to quantify the micronutrient intakes of powerlifters across specific competitive phases and identify any shortcomings in micronutrient intake in comparison to RDA.

### Nutrition practices differ for specific competitive phases

IIFYM/flexible dieting was most often followed during the competition preparation (Fig. 3) and off-season phases (Fig. 5). However, the findings for competition phase (day of competition and the 48 h prior) diet (Fig. 4) indicate a variety of diet strategies, as the frequency of the five most popular diet selections were in the range of 20–40%. In addition, the diet strategies reported for the competition phase contrasted each other, as high and low-calorie diets were reported, and so were high and low (with high protein) CHO diets (Fig. 4). Females more often reported than males implementing dietary approaches which were almost exclusively (90%) implemented to lose weight/body composition goals (Fig. 4), such as restricted/low calorie or high protein with low CHO diets. There were also competitive calibre sub-group differences, as powerlifters with 80 + IPF points more often than 79- IPF points reported IIFYM/flexible dieting and/or a restricted energy diet during the competition period (Fig. 4). The results suggest that powerlifters following a high CHO and/or energy diet prioritise training performance in the days before competition, those following a restricted energy or high protein, low CHO diet prioritise weight loss/body composition, and those following IIFYM/flexible dieting perceive that this diet allows for them to maintain training performance and meet their weight loss/body composition goals. It should be noted that calorie restriction (possibly paired with fasting) and body water storage manipulation are common strategies used by powerlifters for inducing acute weight loss prior to the weigh-in that occurs on the day of powerlifting competition (2 h before) [9]; thus, the findings for the competition phase in the present study likely capture some of these acute weight loss strategies.

### Energy intake practices differ by sex

Several of the findings from the present study indicate that females more often than males follow diets that restrict energy or do not allow for higher energy intakes. For example, long-term use of very high energy diets was reported less among female powerlifters (Fig. 2) and more often reported following a restricted energy diet during competition preparation (Fig. 3) and competition (Fig. 4) than their male counterparts. The total energy expenditure associated with powerlifting training is likely lower compared to other forms of exercise (e.g., endurance); hence, dietary restriction is likely to feature as the primary strategy for achieving a desired body weight or composition [25]. While dietary restriction is common in weight class restricted sports, it is also frequently accompanied by disordered eating in such athletes [26]. More so, disordered eating behaviour is prevalent in female athletes competing in sports emphasising leanness and weight restricted sports [27], and female athletes more often report symptoms of disordered eating than males [28]. Given the weight-class categorisation of powerlifting and that body leanness and weight are key competition performance variables, powerlifting athletes may be at more of a risk of developing disordered eating behaviours.

Despite the likelihood that powerlifters are susceptible to disordered eating, there is minimal scientific inquiry investigating disordered eating in powerlifters (and other strength sports). In a qualitative analysis of disordered eating in competitive female powerlifters (n = 17), weight cutting behaviours were common and associated with disordered eating [29]. The weight class element of powerlifting may present a paradox for some female powerlifters, in which the desire to increase muscle mass (to aid performance) must be balanced against societal ideals for the female body (e.g., to decrease body weight and/or weight class) [29]. Indeed, in previous research, female athletes frequently diet to improve appearance, which was contrasted by males, who most often reported dieting for performance outcomes [30]. Future research is necessary to further elucidate the prevalence of disordered eating behaviours and eating disorders amongst powerlifters (including males [31]).

One possible consequence of disordered eating behaviours is low energy availability (defined as: EI – energy expenditure/FFM), which can occur without disordered eating behaviours (especially in high energy expenditure athletes) [32], but is often underpinned by disordered eating behaviours in female [28, 33] and male athletes [28, 34]. Impaired energy availability could potentially lead to adverse outcomes (e.g., reproductive and skeletal health) such as those included in the Female Athlete Triad [35] and Relative Energy Deficiency in Sports [36], that could foremost negatively affect general health, and training performance, recovery, and adaptation. A prolonged EI below an approximate threshold of < 30 kcal/kg of FFM/day is considered detrimental to physiological function in female athletes [37], although it is less clear whether this threshold is applicable to male athletes [38]. Importantly, a powerlifting athlete may be weight stable and at energy balance but be in a state of low energy availability and experiencing the associated symptoms due to suppressed physiological function [39]. Conversely, adequate EI to support physiological function may need to reach or exceed 45 kcal/kg of FFM/day in some cases [39]. Notably, while these recommendations provide a useful target for EI, a reliable estimation of dietary intake and body composition is needed to accurately implement them and assistance from a sport nutrition or dietetic and/or exercise physiology professional is recommended [35]. Dietary intake of powerlifting athletes could be assessed using dietary logs and food frequency questionnaires, but can be subject to error from underreporting, modified intake caused by and during the measurement period, and inaccurate portion size estimation [40]. In athletic populations, body composition can be estimated with specialised technologies (e.g., dual x-ray absorptiometry, bioelectrical impedance) and field-based measures such as skinfolds [41]. Aided with this information, current recommendations are for physically active women to consume at least 45 kcal/kg of FFM per day to ensure adequate energy availability for physiological function, and between 30 and 45 kcal/kg of FFM per day during periods of intentional weight loss [25, 35].

The source of information informing nutrition practices should also be considered in the context of disordered eating behaviours. In the present study, the coach was the most often cited in-person source of information and females more often than males reported their coach as the source of information to inform hard training day nutrition practices. In previous research, coaches were identified as a prevalent source of information ahead of nutrition practitioners [42]. The coaching environment can increase or reduce risk of eating disorder [43]; thus, it may be beneficial for nutrition practitioners to educate powerlifting coaches on the importance of energy availability, the symptoms of low energy availability, and potential low energy availability management strategies (including the use of a multi-disciplinary team e.g., sport nutrition and mental health practitioners).

### Harder versus easier training day energy periodisation

The energy cost associated with resistance training increases with greater training volumes and exercises that recruit more muscle mass [44]. Thus, athletes may modify their daily EI in accordance with the training demands on the day. For example, decreasing EI on lower volume training days or rest days may better manage EI during the weight cut before competition. Conversely, such a strategy may allow for an increased EI on harder, higher volume training days where the energetic demands of training are higher. Indeed, these strategies were observable in the present population as most powerlifters (71.8%, n = 219) reported intentionally and purposely eating more food/calories on all harder training days with higher training load the most often cited reason for this practice (87.6%, n = 192), compared to normal dietary intake. In contrast, most powerlifters reported not lowering their food/calorie intake on rest/easier training days compared to normal dietary intake (61%, n = 186) (Fig. 7). Strategies to estimate EI typically include dietary logs, questionnaires, and using smart phone dietary tracking applications [45]. While such estimates may contain notable error [40], they are likely preferred to making no such estimation. Another simple, less precise approach with potential utility is estimating energy expenditure from resistance training with the Metabolic Equivalent of Task method and the associated 2011 Compendium of Physical Activities [46]. When doing so, the energy expenditure of an activity in kilocalories = metabolic equivalent of the task x body weight in kilograms x duration of the activity in hours. Taken together, athletes and practitioners have several energy expenditure estimation tools at their disposal that, while limited, allow powerlifting athletes to tailor their EI to the energetic demands of their training.

### Limitations

There are several limitations to the current study. Firstly, our analysis relies on self-reported nutrition practices and the questionnaire described diet practices without defining concepts (e.g., IIFYM) or specific amounts required (e.g., what constitutes ‘high’ or ‘low’ CHO). It is unknown how much of a difference there is between reported dietary trends (e.g., high or low intakes) and actual intake in the current study, since previous work in endurance athletes has observed some discrepancies (up to ~ 35%) [47, 48]. These discrepancies may be explained by participant bias or because responses may reflect what the participants strive or perceive to achieve, rather than actual intake. In the present survey, participants were required to be at least 18 years old to be eligible for inclusion, which excluded younger sub-junior powerlifters (14–17 years old). While sub-junior (at least 18 years old) and junior (19–23 years old) powerlifters were pooled in the analysis, the findings may not generalise to sub-junior powerlifting competitors less than 18 years old. In addition, only 21% of participants (n = 65) were female, which may limit the generalisability of the results. Lastly, the large, global sample size in the current study enabled an exploration of nutrition practices across competitive calibre, and weight, sex, and age classes. However, since most participants (84%) in this study were from North America and Europe, the findings may not be representative of powerlifters in other regions. Various factors, including cultural and socioeconomic influences, among many others, could contribute to differences in food choice [49].

## Conclusion

The current study characterises the general nutrition practices of competitive powerlifters. The key findings were that an IIFYM/flexible dieting approach is popularly used by powerlifters across the competitive cycle and where a special dietary approach was introduced for the competition preparation and off-season phases. The dietary approach chosen by powerlifters for the competitive cycle or competitive phase was not informed by a specific source of information. The findings of the present study indicate a trend in which female powerlifters are more cautious of EI than male counterparts. However, it is not clear whether this finding is connected to disordered eating behaviours, and little is known about the overall prevalence of disordered eating behaviours, and low energy availability, in powerlifters. Most competitive powerlifters reported eating more food/calories on harder training days to fuel for the higher training load. On the other hand, most competitive powerlifters reported not intentionally eating less on rest/easier training days, for the purpose of using the extra calories for muscle growth or recovery. Harder and easier training day nutrition practices were more commonly informed by a specific source of information, with having read/watched it somewhere, coaches, and sports nutritionists most often cited. Finally, powerlifters demonstrate an understanding of the need to achieve adequate fuelling to meet higher training demands by increasing EI on higher volume/intensity days but refrain from reducing EI on rest/easier training days where the training load is lower.

### Supplementary Information

Below is the link to the electronic supplementary material.

Supplementary file1 (DOCX 110 KB)

Supplementary file2 (DOCX 19 KB)

## Data Availability

The dataset associated with this article is available from the corresponding author upon request.

## References

[1] Hughes DC, Ellefsen S, Baar K (2018). Adaptations to endurance and strength training. Cold Spring Harb Perspect Med.

[2] Ye X, Loenneke J, Fahs C (2013). Relationship between lifting performance and skeletal muscle mass in elite powerlifters. J Sports Med Phys Fitness.

[3] Jeukendrup AE (2017). Periodized nutrition for athletes. Sports Med.

[4] Mota JA, Nuckols G, Smith-Ryan AE (2019). Nutritional periodization: applications for the strength athlete. Strength Cond J.

[5] Haff GG, Triplett NT (2015). Essentials of strength training and conditioning.

[6] Slater GJ, Dieter BP, Marsh DJ (2019). Is an energy surplus required to maximize skeletal muscle hypertrophy associated with resistance training. Front Nutr.

[7] Travis SK, Pritchard HJ, Mujika I (2021). Characterizing the tapering practices of United States and Canadian raw powerlifters. J Strength Cond Res.

[8] Nolan D, Lynch AE, Egan B (2022). Self-reported prevalence, magnitude, and methods of rapid weight loss in male and female competitive powerlifters. J Strength Cond Res.

[9] Kwan K, Helms E (2022). Prevalence, magnitude, and methods of weight cutting used by world class powerlifters. J Strength Cond Res.

[10] Slater G, Phillips SM (2013). Nutrition guidelines for strength sports: sprinting, weightlifting, throwing events, and bodybuilding. J Sports Sci.

[11] Morton RW, Murphy KT, McKellar SR (2018). A systematic review, meta-analysis and meta-regression of the effect of protein supplementation on resistance training-induced gains in muscle mass and strength in healthy adults. Br J Sports Med.

[12] Bird S (2010). Strength nutrition: maximizing your anabolic potential. Strength Cond J.

[13] King A, Helms E, Zinn C (2022). The ergogenic effects of acute carbohydrate deeding on resistance exercise performance: a systematic review and meta-analysis. Sports Med.

[14] Ismaeel A, Weems S, Willoughby DS (2018). A comparison of the nutrient intakes of macronutrient-based dieting and strict dieting bodybuilders. Int J Sport Nutr Exerc Metab.

[15] Helms ER, Prnjak K, Linardon J (2019). Towards a sustainable nutrition paradigm in physique sport: a narrative review. Sports.

[16] Westenhoefer J, Stunkard AJ, Pudel V (1999). Validation of the flexible and rigid control dimensions of dietary restraint. Int J Eat Disord.

[17] Oliver JM, Mardock MA, Biehl AJ (2010). Macronutrient intake in collegiate powerlifters participating in off season training. J Int Soc Sports Nutr.

[18] Eysenbach G (2004). Improving the quality of web surveys:the checklist for reporting results of internet E-surveys (CHERRIES). J Med Internet Res.

[19] Heikura IA, Stellingwerff T, Burke LM (2018). Self-reported periodization of nutrition in elite female and male runners and race walkers. Front Physiol.

[20] Westenhoefer J (1991). Dietary restraint and disinhibition: is restraint a homogeneous construct?. Appetite.

[21] Meule A, Westenhöfer J, Kübler A (2011). Food cravings mediate the relationship between rigid, but not flexible control of eating behavior and dieting success. Appetite.

[22] Helms ER, Aragon AA, Fitschen PJ (2014). Evidence-based recommendations for natural bodybuilding contest preparation: nutrition and supplementation. J Int Soc Sports Nutr.

[23] Sim M, Garvican-Lewis LA, Cox GR (2019). Iron considerations for the athlete: a narrative review. Eur J Appl Physiol.

[24] Castell LM, Nieman DC, Bermon S (2019). Exercise-induced illness and inflammation: can immunonutrition and iron help?. Int J Sport Nutr Exerc Metab.

[25] Loucks AB, Kiens B, Wright HH (2013). Energy availability in athletes. J Sports Sci.

[26] Sundgot-Borgen J, Garthe I (2011). Elite athletes in aesthetic and Olympic weight-class sports and the challenge of body weight and body compositions. J Sports Sci.

[27] Torstveit M, Rosenvinge J, Sundgot-Borgen J (2008). Prevalence of eating disorders and the predictive power of risk models in female elite athletes: a controlled study. Scand J Med Sci Sports.

[28] Sundgot-Borgen J, Torstveit MK (2004). Prevalence of eating disorders in elite athletes is higher than in the general population. Clin J Sport Med.

[29] Vargas MLFP, Winter S (2021). Weight on the bar vs. weight on the scale: a qualitative exploration of disordered eating in competitive female powerlifters. Psychol Sport Exerc.

[30] Martinsen M, Bratland-Sanda S, Eriksson AK (2010). Dieting to win or to be thin? A study of dieting and disordered eating among adolescent elite athletes and non-athlete controls. Br J Sports Med.

[31] Eichstadt M, Luzier J, Cho D (2020). Eating disorders in male athletes. Sports Health.

[32] Stellingwerff T, Heikura IA, Meeusen R (2021). Overtraining syndrome (OTS) and relative energy deficiency in sport (RED-S): Shared pathways, symptoms and complexities. Sports Med (Auckland, NZ)..

[33] Gibbs JC, Williams NI, De Souza MJ (2013). Prevalence of individual and combined components of the female athlete triad. Med Sci Sports Exerc.

[34] Chatterton JM, Petrie TA (2013). Prevalence of disordered eating and pathogenic weight control behaviors among male collegiate athletes. Eat Disord.

[35] De Souza MJ, Nattiv A, Joy E (2014). 2014 female athlete triad coalition consensus statement on treatment and return to play of the female athlete triad. Br J Sports Med.

[36] Mountjoy M, Sundgot-Borgen J, Burke L (2018). International Olympic Committee (IOC) consensus statement on relative energy deficiency in sport (RED-S): 2018 update. Int J Sport Nutr Exerc Metab.

[37] Loucks AB, Thuma JR (2003). Luteinizing hormone pulsatility is disrupted at a threshold of energy availability in regularly menstruating women. J Clin Endocrinol Metab.

[38] Burke LM, Close GL, Lundy B (2018). Relative energy deficiency in sport in male athletes: a commentary on its presentation among selected groups of male athletes. Int J Sport Nutr Exerc Metab.

[39] Nattiv A, Loucks AB, Manore MM (2007). American college of sports medicine position stand. The female athlete triad. Med Sci Sports Exerc.

[40] Burke LM, Lundy B, Fahrenholtz IL (2018). Pitfalls of conducting and interpreting estimates of energy availability in free-living athletes. Int J Sport Nutr Exerc Metab.

[41] Meyer NL, Sundgot-Borgen J, Lohman TG (2013). Body composition for health and performance: a survey of body composition assessment practice carried out by the ad hoc research working group on body composition, health and performance under the auspices of the IOC medical commission. Br J Sports Med.

[42] Artioli GG, Gualano B, Franchini E (2010). Prevalence, magnitude, and methods of rapid weight loss among judo competitors. Med Sci Sports Exerc.

[43] Biesecker AC, Martz DM (1999). Impact of coaching style on vulnerability for eating disorders: an analog study. Eat Disord.

[44] Meirelles C, Gomes P (2004). Acute effects of resistance exercise on energy expenditure: revisiting the impact of the training variables. Rev Bras de Medicina do Esporte.

[45] Lieffers JRL, Hanning RM (2012). Dietary assessment and self-monitoring: with nutrition applications for mobile devices. Can J Diet Pract Res.

[46] Ainsworth BE, Haskell WL, Herrmann SD (2011). 2011 compendium of physical activities: a second update of codes and MET values. Med Sci Sports Exerc.

[47] Heikura IA, Burke LM, Mero AA (2017). Dietary microperiodization in elite female and male runners and race walkers during a block of high intensity precompetition training. Int J Sport Nutr Exerc Metab.

[48] Heikura IA, Stellingwerff T, Mero AA (2017). A mismatch between athlete practice and current sports nutrition guidelines among elite female and male middle-and long-distance athletes. Int J Sport Nutr Exerc Metab.

[49] Birkenhead KL, Slater G (2015). A review of factors influencing athletes’ food choices. Sports Med.

