# Supplementary material for: The general nutrition practices of competitive powerlifters vary by competitive calibre and sex, weight, and age class
Source: Eur J Nutr. 2023 Aug 16;62(8):3297–310. doi: 10.1007/s00394-023-03233-6 (PMC10611852; doi:10.1007/s00394-023-03233-6)
Supplement: Supplementary file 1 — Supplementary file1 (DOCX 110 KB) [file 394_2023_3233_MOESM1_ESM.docx]

**Title**

The general nutrition practices of competitive powerlifters vary by competitive calibre and sex, weight, and age class

**Authors**

Andrew King^1^, Kedric Kwan^1^, Ivan Jukic^1,2^, Caryn Zinn^1^, and Eric Helms^1^

**Affiliations**

^1^Sport Performance Research Institute New Zealand (SPRINZ), Auckland University of Technology, Auckland, New Zealand

^2^School of Engineering, Computer and Mathematical Sciences, Auckland University of Technology, Auckland, New Zealand

**ORCID**

Andrew King: https://orcid.org/0000-0002-9332-6506

Ivan Jukic: https://orcid.org/0000-0002-0900-9410

**Corresponding Author**

Andrew King

PhD Candidate

Sport Performance Research Institute New Zealand (SPRINZ)

Auckland University of Technology

17 Antares Place, Mairangi Bay

Auckland, New Zealand, 0632

Email: andrew.king@aut.ac.nz

Supplementary File I

Peri-workout Nutrition Practices of Powerlifters

Start of Block: General Descriptive and Training History

Q1.1 In which powerlifting division do you compete?

- Female (1)
- Male (2)

| 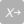 |
| --- |

Q1.2 In which country do you currently reside?

▼ Afghanistan (1) ... Zimbabwe (1357)

Q1.3 In which age class do you currently compete?

- Sub-junior (16-18 years) (1)
- Junior (19-23 years) (2)
- Open (24-39 years) (3)
- Masters (40+ years) (4)

Q1.4 In which weight class do you typically compete?

- 43 kg (sub-junior and junior women only) (1)
- 47 kg (women) (2)
- 52 kg (women) (3)
- 53 kg (sub-junior and junior men only) (4)
- 57 kg (women) (5)
- 59 kg (men) (6)
- 63 kg (women) (7)
- 66 kg (men) (8)
- 72 kg (women) (9)
- 74 kg (men) (10)
- 83 kg (men) (11)
- 84 kg (women) (12)
- 84+ kg (women) (13)
- 93 kg (men) (14)
- 105 kg (men) (15)
- 120 kg (men) (16)
- 120+ kg (men) (17)

Q1.5 What is the highest level of powerlifting competition you have competed at?

- Club (1)
- Regional (2)
- National (3)
- International (4)

Q1.6 Within what range is your best IPF points score (three lift competition total only)? **(Please calculate using: https://www.ipfpointscalculator.com/)**

- Less than 50 (15)
- 50 – 54 (12)
- 55 – 59 (13)
- 60 – 64 (14)
- 65 – 69 (9)
- 70 – 74 (10)
- 75 – 79 (1)
- 80 – 84 (2)
- 85 – 89 (3)
- 90 – 94 (4)
- 95 – 99 (5)
- 100 – 104 (6)
- 105 – 109 (7)
- Greater than 110 (8)

Q1.7 How many years total have you been resistance training?

- 0 – 1 years (1)
- 1 – 2 years (2)
- 2 – 3 years (3)
- 3 – 4 years (4)
- 4 – 5 years (5)
- 6 – 7 years (6)
- 7 – 8 years (7)
- 8 – 9 years (8)
- 9 – 10 years (9)
- 10+ years (10)

Q1.8 How many years of dedicated powerlifting training/competition experience? **(This refers to the time you have specifically trained the squat, bench press and deadlift for the purpose of increasing the 1-repetition maximum of these lifts)**

- 0 – 1 years (1)
- 1 – 2 years (2)
- 2 – 3 years (3)
- 3 – 4 years (4)
- 4 – 5 years (5)
- 5 – 6 years (6)
- 6 – 7 years (7)
- 7 – 8 years (8)
- 8 – 9 years (9)
- 9 – 10 years (10)
- 10+ years (11)

End of Block: General Descriptive and Training History

Start of Block: Nutrition for Competitive Cycle

Q2.1 Do you deliberately and consistently follow a long-term unique/special dietary plan to support your training goals (i.e. all year round during all training phases)? 
**(Tick all options that are important in describing what you do)**

- Vegan/vegetarian (1)
- Paleo (2)
- Paleo for athletes (Paleo but with more carbohydrate around training sessions) (3)
- If It Fits Your Macros (IIFYM) or flexible dieting (4)
- Low carb, high fat (LCHF) or ketogenic (5)
- High protein, low carb (6)
- Gluten free (7)
- High carb (8)
- Very high energy/extra energy (high calorie) (9)
- Restricted energy (low calorie) (13)
- FODMAP (10)
- Other: Please state. (11) ________________________________________________
- ⊗No, I do not follow any unique/special dietary plan (12)

Display This Question:

If Do you deliberately and consistently follow a long-term unique/special dietary plan to support yo... = Vegan/vegetarian

Q2.2 What is the main purpose of following the vegan/vegetarian long-term diet?  **(Tick all options that are important in describing what you do)**

- Religious/cultural reasons (1)
- Ethical/moral reasons (2)
- To achieve better quality training overall (e.g. performance) (3)
- To enhance muscle growth or recovery (4)
- To lose weight/body composition goals (5)
- Better health (6)
- Diagnosed allergies or intolerances (7)
- I enjoy aspects of this diet (e.g. the foods, variety, or flexibility involved) (10)
- A source of information (e.g. someone told you or you read/watched it somewhere) told me to follow this diet (9)
- Other: Please specify (8) ________________________________________________

Display This Question:

If What is the main purpose of following the vegan/vegetarian long-term diet?  (Tick all options tha... = A source of information (e.g. someone told you or you read/watched it somewhere) told me to follow this diet

Q2.3
What is the source of information (e.g. someone told you or you read/watched it somewhere) that informs your practice of following a vegan/vegetarian diet long-term? 
(Tick all options that are relevant to informing your practice)

- Coach (1)
- Sport nutritionist (2)
- Dietician (3)
- Medical Doctor (4)
- Physiotherapist (5)
- Family Member (6)
- A friend (7)
- Scientist (8)
- Training partner (9)
- Personal trainer (11)
- I read/watched it somewhere (e.g. blog, journal article, or Youtube etc.) (10)
- Other: Please state. (12) ________________________________________________

Display This Question:

If Do you deliberately and consistently follow a long-term unique/special dietary plan to support yo... = Paleo

Q2.4 What is the main purpose of following a Paleo diet long-term? (Tick all options that are important in describing what you do)

- Religious/cultural reasons (1)
- Ethical/moral reasons (4)
- To achieve better quality training overall (e.g. performance) (5)
- To enhance muscle growth or recovery (6)
- To lose weight/body composition goals (7)
- Better health (8)
- Diagnosed allergies or intolerances (9)
- I enjoy aspects of this diet (e.g. the foods, variety, or flexibility involved) (12)
- A source of information (e.g. someone told you or you read/watched it somewhere) told me to follow this diet (10)
- Other: Please specify (11) ________________________________________________

Display This Question:

If What is the main purpose of following a Paleo diet long-term? (Tick all options that are importan... = A source of information (e.g. someone told you or you read/watched it somewhere) told me to follow this diet

Q2.5 What is the source of information (e.g. someone told you or you read/watched it somewhere) that informs your practice of following a Paleo diet long-term? (Tick all options that are relevant to informing your practice)

- Coach (1)
- Sport nutritionist (4)
- Dietician (5)
- Medical Doctor (6)
- Physiotherapist (7)
- Family Member (8)
- A friend (9)
- Scientist (10)
- Training partner (11)
- Personal trainer (12)
- I read/watched it somewhere (e.g. blog, journal article, or Youtube etc.) (13)
- Other: Please state. (14) ________________________________________________

Display This Question:

If Do you deliberately and consistently follow a long-term unique/special dietary plan to support yo... = Paleo for athletes (Paleo but with more carbohydrate around training sessions)

Q2.6 What is the main purpose of following a Paleo for athletes diet long-term? (Tick all options that are important in describing what you do)

- Religious/cultural reasons (1)
- Ethical/moral reasons (4)
- To achieve better quality training overall (e.g. performance) (5)
- To enhance muscle growth or recovery (6)
- To lose weight/body composition goals (7)
- Better health (8)
- Diagnosed allergies or intolerances (9)
- I enjoy aspects of this diet (e.g. the foods, variety, or flexibility involved) (12)
- A source of information (e.g. someone told you or you read/watched it somewhere) told me to follow this diet (10)
- Other: Please specify (11) ________________________________________________

Display This Question:

If What is the main purpose of following a Paleo for athletes diet long-term? (Tick all options that... = A source of information (e.g. someone told you or you read/watched it somewhere) told me to follow this diet

Q2.7 What is the source of information (e.g. someone told you or you read/watched it somewhere) that informs your practice of following a Paleo for athletes diet long-term? (Tick all options that are relevant to informing your practice)

- Coach (1)
- Sport nutritionist (4)
- Dietician (5)
- Medical Doctor (6)
- Physiotherapist (7)
- Family Member (8)
- A friend (9)
- Scientist (10)
- Training partner (11)
- Personal trainer (12)
- I read/watched it somewhere (e.g. blog, journal article, or Youtube etc.) (13)
- Other: Please state. (14) ________________________________________________

Display This Question:

If Do you deliberately and consistently follow a long-term unique/special dietary plan to support yo... = If It Fits Your Macros (IIFYM) or flexible dieting

Q2.8 What is the main purpose of following If It Fits Your Macros (IIFYM) or flexible dieting long-term? 
(Tick all options that are important in describing what you do)

- Religious/cultural reasons (1)
- Ethical/moral reasons (4)
- To achieve better quality training overall (e.g. performance) (5)
- To enhance muscle growth or recovery (6)
- To lose weight/body composition goals (7)
- Better health (8)
- Diagnosed allergies or intolerances (9)
- I enjoy aspects of this diet (e.g. the foods, variety, or flexibility involved) (12)
- A source of information (e.g. someone told you or you read/watched it somewhere) told me to follow this diet (10)
- Other: Please specify (11) ________________________________________________

Display This Question:

If What is the main purpose of following If It Fits Your Macros (IIFYM) or flexible dieting long-ter... = A source of information (e.g. someone told you or you read/watched it somewhere) told me to follow this diet

Q2.9 What is the source of information (e.g. someone told you or you read/watched it somewhere) that informs your practice of following If It Fits Your Macros (IIFYM) or flexible dieting long-term? 
(Tick all options that are relevant to informing your practice)

- Coach (1)
- Sport nutritionist (4)
- Dietician (5)
- Medical Doctor (6)
- Physiotherapist (7)
- Family Member (8)
- A friend (9)
- Scientist (10)
- Training partner (11)
- Personal trainer (12)
- I read/watched it somewhere (e.g. blog, journal article, or Youtube etc.) (13)
- Other: Please state. (14) ________________________________________________

Display This Question:

If Do you deliberately and consistently follow a long-term unique/special dietary plan to support yo... = Low carb, high fat (LCHF) or ketogenic

Q2.10 What is the main purpose of following a low carb, high fat (LCHF) or ketogenic diet long-term? 
(Tick all options that are important in describing what you do)

- Religious/cultural reasons (1)
- Ethical/moral reasons (4)
- To achieve better quality training overall (e.g. performance) (5)
- To enhance muscle growth or recovery (6)
- To lose weight/body composition goals (7)
- Better health (8)
- Diagnosed allergies or intolerances (9)
- I enjoy aspects of this diet (e.g. the foods, variety, or flexibility involved) (12)
- A source of information (e.g. someone told you or you read/watched it somewhere) told me to follow this diet (10)
- Other: Please specify (11) ________________________________________________

Display This Question:

If What is the main purpose of following a low carb, high fat (LCHF) or ketogenic diet long-term?  (... = A source of information (e.g. someone told you or you read/watched it somewhere) told me to follow this diet

Q2.11 What is the source of information (e.g. someone told you or you read/watched it somewhere) that informs your practice of following a low carb, high fat (LCHF) or ketogenic diet long-term? 
(Tick all options that are relevant to informing your practice)

- Coach (1)
- Sport nutritionist (4)
- Dietician (5)
- Medical Doctor (6)
- Physiotherapist (7)
- Family Member (8)
- A friend (9)
- Scientist (10)
- Training partner (11)
- Personal trainer (12)
- I read/watched it somewhere (e.g. blog, journal article, or Youtube etc.) (13)
- Other: Please state. (14) ________________________________________________

Display This Question:

If Do you deliberately and consistently follow a long-term unique/special dietary plan to support yo... = High protein, low carb

Q2.12 What is the main purpose of following a high protein, low carb diet long-term? 
(Tick all options that are important in describing what you do)

- Religious/cultural reasons (1)
- Ethical/moral reasons (4)
- To achieve better quality training overall (e.g. performance) (5)
- To enhance muscle growth or recovery (6)
- To lose weight/body composition goals (7)
- Better health (8)
- Diagnosed allergies or intolerances (9)
- I enjoy aspects of this diet (e.g. the foods, variety, or flexibility involved) (12)
- A source of information (e.g. someone told you or you read/watched it somewhere) told me to follow this diet (10)
- Other: Please specify (11) ________________________________________________

Display This Question:

If What is the main purpose of following a high protein, low carb diet long-term?  (Tick all options... = A source of information (e.g. someone told you or you read/watched it somewhere) told me to follow this diet

Q2.13 What is the source of information (e.g. someone told you or you read/watched it somewhere) that informs your practice of following a high protein, low carb diet long-term? 
(Tick all options that are relevant to informing your practice)

- Coach (1)
- Sport nutritionist (4)
- Dietician (5)
- Medical Doctor (6)
- Physiotherapist (7)
- Family Member (8)
- A friend (9)
- Scientist (10)
- Training partner (11)
- Personal trainer (12)
- I read/watched it somewhere (e.g. blog, journal article, or Youtube etc.) (13)
- Other: Please state. (14) ________________________________________________

Display This Question:

If Do you deliberately and consistently follow a long-term unique/special dietary plan to support yo... = Gluten free

Q2.14 What is the main purpose of following a gluten free diet long-term? 
(Tick all options that are important in describing what you do)

- Religious/cultural reasons (1)
- Ethical/moral reasons (4)
- To achieve better quality training overall (e.g. performance) (5)
- To enhance muscle growth or recovery (6)
- To lose weight/body composition goals (7)
- Better health (8)
- Diagnosed allergies or intolerances (9)
- I enjoy aspects of this diet (e.g. the foods, variety, or flexibility involved) (12)
- A source of information (e.g. someone told you or you read/watched it somewhere) told me to follow this diet (10)
- Other: Please specify (11) ________________________________________________

Display This Question:

If What is the main purpose of following a gluten free diet long-term?  (Tick all options that are i... = A source of information (e.g. someone told you or you read/watched it somewhere) told me to follow this diet

Q2.15 What is the source of information (e.g. someone told you or you read/watched it somewhere) that informs your practice of following a gluten free diet long-term? 
(Tick all options that are relevant to informing your practice)

- Coach (1)
- Sport nutritionist (4)
- Dietician (5)
- Medical Doctor (6)
- Physiotherapist (7)
- Family Member (8)
- A friend (9)
- Scientist (10)
- Training partner (11)
- Personal trainer (12)
- I read/watched it somewhere (e.g. blog, journal article, or Youtube etc.) (13)
- Other: Please state. (14) ________________________________________________

Display This Question:

If Do you deliberately and consistently follow a long-term unique/special dietary plan to support yo... = High carb

Q2.16 What is the main purpose of following a high carb diet long-term? 
(Tick all options that are important in describing what you do)

- Religious/cultural reasons (1)
- Ethical/moral reasons (4)
- To achieve better quality training overall (e.g. performance) (5)
- To enhance muscle growth or recovery (6)
- To lose weight/body composition goals (7)
- Better health (8)
- Diagnosed allergies or intolerances (9)
- I enjoy aspects of this diet (e.g. the foods, variety, or flexibility involved) (12)
- A source of information (e.g. someone told you or you read/watched it somewhere) told me to follow this diet (10)
- Other: Please specify (11) ________________________________________________

Display This Question:

If What is the main purpose of following a high carb diet long-term?  (Tick all options that are imp... = A source of information (e.g. someone told you or you read/watched it somewhere) told me to follow this diet

Q2.17 What is the source of information (e.g. someone told you or you read/watched it somewhere) that informs your practice of following a high carb diet long-term? 
(Tick all options that are relevant to informing your practice)

- Coach (1)
- Sport nutritionist (4)
- Dietician (5)
- Medical Doctor (6)
- Physiotherapist (7)
- Family Member (8)
- A friend (9)
- Scientist (10)
- Training partner (11)
- Personal trainer (12)
- I read/watched it somewhere (e.g. blog, journal article, or Youtube etc.) (13)
- Other: Please state. (14) ________________________________________________

Display This Question:

If Do you deliberately and consistently follow a long-term unique/special dietary plan to support yo... = Very high energy/extra energy (high calorie)

Q2.18 What is the main purpose of following a very high energy/extra energy (high calorie) diet long-term? 
(Tick all options that are important in describing what you do)

- Religious/cultural reasons (1)
- Ethical/moral reasons (4)
- To achieve better quality training overall (e.g. performance) (5)
- To enhance muscle growth or recovery (6)
- To lose weight/body composition goals (7)
- Better health (8)
- Diagnosed allergies or intolerances (9)
- I enjoy aspects of this diet (e.g. the foods, variety, or flexibility involved) (12)
- A source of information (e.g. someone told you or you read/watched it somewhere) told me to follow this diet (10)
- Other: Please specify (11) ________________________________________________

Display This Question:

If What is the main purpose of following a very high energy/extra energy (high calorie) diet long-te... = A source of information (e.g. someone told you or you read/watched it somewhere) told me to follow this diet

Q2.19 What is the source of information (e.g. someone told you or you read/watched it somewhere) that informs your practice of following a high energy/extra energy (high calorie) diet long-term? 
(Tick all options that are relevant to informing your practice)

- Coach (1)
- Sport nutritionist (4)
- Dietician (5)
- Medical Doctor (6)
- Physiotherapist (7)
- Family Member (8)
- A friend (9)
- Scientist (10)
- Training partner (11)
- Personal trainer (12)
- I read/watched it somewhere (e.g. blog, journal article, or Youtube etc.) (13)
- Other: Please state. (14) ________________________________________________

Display This Question:

If Do you deliberately and consistently follow a long-term unique/special dietary plan to support yo... = Restricted energy (low calorie)

Q2.20 What is the main purpose of following a restricted energy (low calorie) diet long-term? 
(Tick all options that are important in describing what you do)

- Religious/cultural reasons (1)
- Ethical/moral reasons (4)
- To achieve better quality training overall (e.g. performance) (5)
- To enhance muscle growth or recovery (6)
- To lose weight/body composition goals (7)
- Better health (8)
- Diagnosed allergies or intolerances (9)
- I enjoy aspects of this diet (e.g. the foods, variety, or flexibility involved) (12)
- A source of information (e.g. someone told you or you read/watched it somewhere) told me to follow this diet (10)
- Other: Please specify (11) ________________________________________________

Display This Question:

If What is the main purpose of following a restricted energy (low calorie) diet long-term?  (Tick al... = A source of information (e.g. someone told you or you read/watched it somewhere) told me to follow this diet

Q2.21 What is the source of information (e.g. someone told you or you read/watched it somewhere) that informs your practice of following a restricted energy (low calorie) diet long-term? 
(Tick all options that are relevant to informing your practice)

- Coach (1)
- Sport nutritionist (4)
- Dietician (5)
- Medical Doctor (6)
- Physiotherapist (7)
- Family Member (8)
- A friend (9)
- Scientist (10)
- Training partner (11)
- Personal trainer (12)
- I read/watched it somewhere (e.g. blog, journal article, or Youtube etc.) (13)
- Other: Please state. (14) ________________________________________________

Display This Question:

If Do you deliberately and consistently follow a long-term unique/special dietary plan to support yo... = FODMAP

Q2.22 What is the main purpose of following a FODMAP diet long-term? 
(Tick all options that are important in describing what you do)

- Religious/cultural reasons (1)
- Ethical/moral reasons (4)
- To achieve better quality training overall (e.g. performance) (5)
- To enhance muscle growth or recovery (6)
- To lose weight/body composition goals (7)
- Better health (8)
- Diagnosed allergies or intolerances (9)
- I enjoy aspects of this diet (e.g. the foods, variety, or flexibility involved) (12)
- A source of information (e.g. someone told you or you read/watched it somewhere) told me to follow this diet (10)
- Other: Please specify (11) ________________________________________________

Display This Question:

If What is the main purpose of following a FODMAP diet long-term?  (Tick all options that are import... = A source of information (e.g. someone told you or you read/watched it somewhere) told me to follow this diet

Q2.23 What is the source of information (e.g. someone told you or you read/watched it somewhere) that informs your practice of following a FODMAP diet long-term? 
(Tick all options that are relevant to informing your practice)

- Coach (1)
- Sport nutritionist (4)
- Dietician (5)
- Medical Doctor (6)
- Physiotherapist (7)
- Family Member (8)
- A friend (9)
- Scientist (10)
- Training partner (11)
- Personal trainer (12)
- I read/watched it somewhere (e.g. blog, journal article, or Youtube etc.) (13)
- Other: Please state. (14) ________________________________________________

Display This Question:

If Do you deliberately and consistently follow a long-term unique/special dietary plan to support yo... = Other: Please state.

Q2.24 What is the main purpose of following the long-term diet you have specified (i.e. ${Q2.1/ChoiceTextEntryValue/11})? 
(Tick all options that are important in describing what you do)

- Religious/cultural reasons (1)
- Ethical/moral reasons (4)
- To achieve better quality training overall (e.g. performance) (5)
- To enhance muscle growth or recovery (6)
- To lose weight/body composition goals (7)
- Better health (8)
- Diagnosed allergies or intolerances (9)
- I enjoy aspects of this diet (e.g. the foods, variety, or flexibility involved) (12)
- A source of information (e.g. someone told you or you read/watched it somewhere) told me to follow this diet (10)
- Other: Please specify (11) ________________________________________________

Display This Question:

If What is the main purpose of following the long-term diet you have specified (i.e. ... = A source of information (e.g. someone told you or you read/watched it somewhere) told me to follow this diet

Q2.25 What is the source of information (e.g. someone told you or you read/watched it somewhere) that informs your practice of following  the long-term diet you have specified (i.e. ${Q2.1/ChoiceTextEntryValue/11})? 
(Tick all options that are relevant to informing your practice)

- Coach (1)
- Sport nutritionist (4)
- Dietician (5)
- Medical Doctor (6)
- Physiotherapist (7)
- Family Member (8)
- A friend (9)
- Scientist (10)
- Training partner (11)
- Personal trainer (12)
- I read/watched it somewhere (e.g. blog, journal article, or Youtube etc.) (13)
- Other: Please state. (14) ________________________________________________

End of Block: Nutrition for Competitive Cycle

Start of Block: Nutrition for Competitive Phases

Q3.1 Do you deliberately but periodically introduce a special dietary plan to support your training goals (i.e. periodically following one or several nutrition strategies over the annual training/competition program)?  **(Tick all options that are important in describing what you do)**

 If so, when do you follow a special dietary plan?
 Please refer to the below table for a generalised yearly plan:


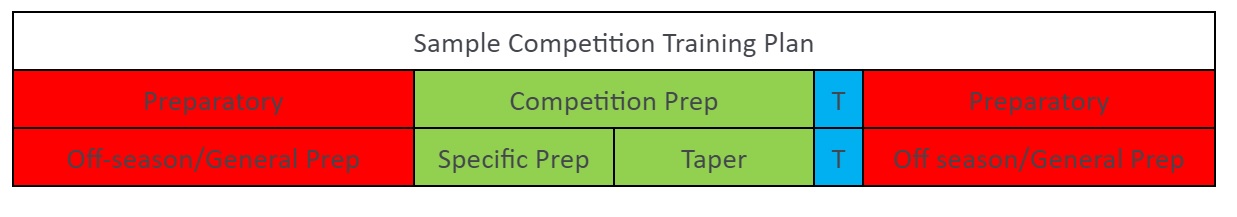


- Off-season/general preparation (2)
- Competition preparation phase (3)
- Competition (including the day of the competition and the 48 hours preceding) (4)
- Transition (immediately post-competition, leading into an off-season or another general preparation) (5)
- Return from injury (6)
- Other time period: Please state. (7) ________________________________________________
- ⊗No, I don’t periodically follow a special dietary plan during the yearly training program (1)

Display This Question:

If Do you deliberately but periodically introduce a special dietary plan to support your training go... = Off-season/general preparation

Q3.2 What special dietary plan do you follow for off-season/general preparation phase? 
**(Tick all options that are important in describing what you do)**

- Vegan/vegetarian (1)
- Paleo (2)
- Paleo for athletes (Paleo but with more carbohydrates around training sessions) (3)
- If It Fits Your Macros (IIFYM) or flexible dieting (4)
- Low carb, high fat (LCHF) or ketogenic (5)
- High protein, low carb (6)
- Gluten free (7)
- High carb (8)
- Very high energy/extra energy (high calorie) (9)
- Restricted energy (low calorie) (12)
- FODMAP (10)
- Other: Please state. (11) ________________________________________________

Display This Question:

If Do you deliberately but periodically introduce a special dietary plan to support your training go... = Competition preparation phase

Q3.3
What special dietary plan do you follow for the competition preparation phase? 
(Tick all options that are important in describing what you do)

- Vegan/vegetarian (1)
- Paleo (2)
- Paleo for athletes (Paleo but with more carbohydrates around training sessions) (3)
- If It Fits Your Macros (IIFYM) or flexible dieting (4)
- Low carb, high fat (LCHF) or ketogenic (5)
- High protein, low carb (6)
- Gluten free (7)
- High carb (8)
- Very high energy/extra energy (high calorie) (9)
- Restricted energy (low calorie) (12)
- FODMAP (10)
- Other: Please state. (11) ________________________________________________

Display This Question:

If Do you deliberately but periodically introduce a special dietary plan to support your training go... = Competition (including the day of the competition and the 48 hours preceding)

Q3.4

What special dietary plan do you follow for the competition phase (including the day of competition and the 48 hours preceding)? 
(Tick all options that are important in describing what you do)

- Vegan/vegetarian (1)
- Paleo (2)
- Paleo for athletes (Paleo but with more carbohydrates around training sessions) (3)
- If It Fits Your Macros (IIFYM) or flexible dieting (4)
- Low carb, high fat (LCHF) or ketogenic (5)
- High protein, low carb (6)
- Gluten free (7)
- High carb (8)
- Very high energy/extra energy (high calorie) (9)
- Restricted energy (low calorie) (12)
- FODMAP (10)
- Other: Please state. (11) ________________________________________________

Display This Question:

If Do you deliberately but periodically introduce a special dietary plan to support your training go... = Transition (immediately post-competition, leading into an off-season or another general preparation)

Q3.5
What special dietary plan do you follow for the transition phase (i.e. immediately post-competition, leading into an off-season or another general preparation)? 

(Tick all options that are important in describing what you do)

- Vegan/vegetarian (1)
- Paleo (2)
- Paleo for athletes (Paleo but with more carbohydrates around training sessions) (3)
- If It Fits Your Macros (IIFYM) or flexible dieting (4)
- Low carb, high fat (LCHF) or ketogenic (5)
- High protein, low carb (6)
- Gluten free (7)
- High carb (8)
- Very high energy/extra energy (high calorie) (9)
- Restricted energy (low calorie) (12)
- FODMAP (10)
- Other: Please state. (11) ________________________________________________

Display This Question:

If Do you deliberately but periodically introduce a special dietary plan to support your training go... = Return from injury

Q3.6
What special dietary plan do you follow for a return from injury? 

(Tick all options that are important in describing what you do)

- Vegan/vegetarian (1)
- Paleo (2)
- Paleo for athletes (Paleo but with more carbohydrates around training sessions) (3)
- If It Fits Your Macros (IIFYM) or flexible dieting (4)
- Low carb, high fat (LCHF) or ketogenic (5)
- High protein, low carb (6)
- Gluten free (7)
- High carb (8)
- Very high energy/extra energy (high calorie) (9)
- Restricted energy (low calorie) (12)
- FODMAP (10)
- Other: Please state. (11) ________________________________________________

Display This Question:

If Do you deliberately but periodically introduce a special dietary plan to support your training go... = Other time period: Please state.

Q3.7
What special dietary plan do you follow for the other time period you have specified (i.e. ${Q3.1/ChoiceTextEntryValue/7}? 

(Tick all options that are important in describing what you do)

- Vegan/vegetarian (1)
- Paleo (2)
- Paleo for athletes (Paleo but with more carbohydrates around training sessions) (3)
- If It Fits Your Macros (IIFYM) or flexible dieting (4)
- Low carb, high fat (LCHF) or ketogenic (5)
- High protein, low carb (6)
- Gluten free (7)
- High carb (8)
- Very high energy/extra energy (high calorie) (9)
- Restricted energy (low calorie) (12)
- FODMAP (10)
- Other: Please state. (11) ________________________________________________

| Page Break |  |
| --- | --- |

Display This Question:

If Do you deliberately but periodically introduce a special dietary plan to support your training go... = Off-season/general preparation

Q3.8 What is the main purpose of following a specific diet during an off-season/general preparation phase? 
If you have selected more than one special dietary plans for this phase **(you chose: ${Q3.2/ChoiceGroup/SelectedChoices})**, please use the "other" text box to specify the purpose for each of these special dietary plans.  **(Tick all options that are important in describing what you do)**

- Religious/cultural reasons (1)
- Ethical/moral reasons (2)
- To achieve better quality training overall (3)
- To enhance muscle growth or recovery from training generally (4)
- To lose weight/body composition goals (5)
- Better health (6)
- Diagnosed allergies or intolerances (7)
- I enjoy aspects of this diet (e.g. the foods, variety, or flexibility involved) (10)
- An information source (e.g. someone told you or you read/watched it somewhere) told me to follow this diet (9)
- Other: Please specify (8) ________________________________________________

Display This Question:

If What is the main purpose of following a specific diet during an off-season/general preparation ph... = An information source (e.g. someone told you or you read/watched it somewhere) told me to follow this diet

Q3.9 What information source told you to follow this diet for the off-season/general preparation phase? **(Tick all options that are relevant to informing your practice)**

- Coach (1)
- Sport nutritionist (4)
- Dietician (5)
- Medical Doctor (6)
- Physiotherapist (7)
- Family Member (8)
- A friend (9)
- Scientist (10)
- Training partner (11)
- Personal trainer (13)
- I read/watched it somewhere (e.g. blog, journal article, or Youtube etc.) (12)
- Other: Please state. (14) ________________________________________________

Display This Question:

If Do you deliberately but periodically introduce a special dietary plan to support your training go... = Competition preparation phase

Q3.10 What is the main purpose of following a specific diet during a competition preparation phase? 


If you have selected more than one special dietary plans for this phase (you chose: ${Q3.3/ChoiceGroup/SelectedChoices}), please use the "other" text box to specify the purpose for each of these special dietary plans. 


(Tick all options that are important in describing what you do)

- Religious/cultural reasons (1)
- Ethical/moral reasons (2)
- To achieve better quality training overall (e.g. performance) (3)
- To enhance muscle growth or recovery (4)
- To lose weight/body composition goals (5)
- Better health (6)
- Diagnosed allergies or intolerances (7)
- I enjoy aspects of this diet (e.g. the foods, variety, or flexibility involved) (10)
- An information source (e.g. someone told you or you read/watched it somewhere) told me to follow this diet (9)
- Other: Please specify (8) ________________________________________________

Display This Question:

If What is the main purpose of following a specific diet during a competition preparation phase?  If... = An information source (e.g. someone told you or you read/watched it somewhere) told me to follow this diet

Q3.11 What information source told you to follow this diet for the competition preparation phase? **(Tick all options that are relevant to informing your practice)**

- Coach (1)
- Sport nutritionist (12)
- Dietician (13)
- Medical Doctor (14)
- Physiotherapist (15)
- Family Member (16)
- A friend (17)
- Scientist (18)
- Training partner (19)
- Personal trainer (21)
- I read/watched it somewhere (e.g. blog, journal article, or Youtube etc.) (20)
- Other: Please state. (22) ________________________________________________

Display This Question:

If Do you deliberately but periodically introduce a special dietary plan to support your training go... = Competition (including the day of the competition and the 48 hours preceding)

Q3.12 What is the main purpose of following a specific diet during a competition phase (i.e. day of competition and the 48 hours preceding)? 


If you have selected more than one special dietary plans for this phase (you chose: ${Q3.4/ChoiceGroup/SelectedChoices}), please use the "other" text box to specify the purpose for each of these special dietary plans. 
 
(Tick all options that are important in describing what you do)

- Religious/cultural reasons (1)
- Ethical/moral reasons (2)
- To achieve better quality training overall (3)
- To enhance muscle growth or recovery from training generally (4)
- To lose weight/body composition goals (5)
- Better health (6)
- Diagnosed allergies or intolerances (7)
- I enjoy aspects of this diet (e.g. the foods, variety, or flexibility involved) (10)
- An information source (e.g. someone told you or you read/watched it somewhere) told me to follow this diet (9)
- Other: Please specify (8) ________________________________________________

Display This Question:

If What is the main purpose of following a specific diet during a competition phase (i.e. day of com... = An information source (e.g. someone told you or you read/watched it somewhere) told me to follow this diet

Q3.13 What information source told you to follow this diet for the competition phase? (i.e. the day of competition and the 48 hours preceding)? (Tick all options that are relevant to informing your practice)

- Coach (1)
- Sport nutritionist (4)
- Dietician (5)
- Medical Doctor (6)
- Physiotherapist (7)
- Family Member (8)
- A friend (9)
- Scientist (10)
- Training partner (11)
- Personal trainer (13)
- I read/watched it somewhere (e.g. blog, journal article, or Youtube etc.) (12)
- Other: Please state. (14) ________________________________________________

Display This Question:

If Do you deliberately but periodically introduce a special dietary plan to support your training go... = Transition (immediately post-competition, leading into an off-season or another general preparation)

Q3.14 What is the main purpose of following a specific diet during a transition phase (immediately post-competition, leading into an off-season or another general preparation)? 


If you have selected more than one special dietary plans for this phase (you chose: ${Q3.5/ChoiceGroup/SelectedChoices}), please use the "other" text box to specify the purpose for each of these special dietary plans. 

 **(Tick all options that are important in describing what you do)**

- Religious/cultural reasons (1)
- Ethical/moral reasons (2)
- To achieve better quality training overall (3)
- To enhance muscle growth or recovery from training generally (4)
- To lose weight/body composition goals (5)
- Better health (6)
- Diagnosed allergies or intolerances (7)
- I enjoy aspects of this diet (e.g. the foods, variety, or flexibility involved) (10)
- An information source (e.g. someone told you or you read/watched it somewhere) told me to follow this diet (9)
- Other: Please specify (8) ________________________________________________

Display This Question:

If What is the main purpose of following a specific diet during a transition phase (immediately post... = An information source (e.g. someone told you or you read/watched it somewhere) told me to follow this diet

Q3.15 What information source told you to follow this diet for a transition phase? (Tick all options that are relevant to informing your practice)

- Coach (1)
- Sport nutritionist (4)
- Dietician (5)
- Medical Doctor (6)
- Physiotherapist (7)
- Family Member (8)
- A friend (9)
- Scientist (10)
- Training partner (11)
- Personal trainer (13)
- I read/watched it somewhere (e.g. blog, journal article, or Youtube etc.) (12)
- Other: Please state. (14) ________________________________________________

Display This Question:

If Do you deliberately but periodically introduce a special dietary plan to support your training go... = Return from injury

Q3.16 What is the main purpose of following a specific diet during a return from injury? 


If you have selected more than one special dietary plans for this phase (you chose: ${Q3.6/ChoiceGroup/SelectedChoices}), please use the "other" text box to specify the purpose for each of these special dietary plans. 


**(Tick all options that are important in describing what you do)**

- Religious/cultural reasons (1)
- Ethical/moral reasons (2)
- To achieve better quality training overall (3)
- To enhance muscle growth or recovery from training generally (4)
- To lose weight/body composition goals (5)
- Better health (6)
- Diagnosed allergies or intolerances (7)
- I enjoy aspects of this diet (e.g. the foods, variety, or flexibility involved) (10)
- An information source (e.g. someone told you or you read/watched it somewhere) told me to follow this diet (9)
- Other: Please specify (8) ________________________________________________

Display This Question:

If What is the main purpose of following a specific diet during a return from injury?  If you have s... = An information source (e.g. someone told you or you read/watched it somewhere) told me to follow this diet

Q3.17 What information source told you to follow this diet during a return from injury? **(Tick all options that are relevant to informing your practice)**

- Coach (1)
- Sport nutritionist (4)
- Dietician (5)
- Medical Doctor (6)
- Physiotherapist (7)
- Family Member (8)
- A friend (9)
- Scientist (10)
- Training partner (11)
- Personal trainer (13)
- I read/watched it somewhere (e.g. blog, journal article, or Youtube etc.) (12)
- Other: Please state. (14) ________________________________________________

Display This Question:

If Do you deliberately but periodically introduce a special dietary plan to support your training go... = Other time period: Please state.

Q3.18 What is the main purpose of following a specific diet during ${Q3.1/ChoiceTextEntryValue/7}? 


If you have selected more than one special dietary plans for this phase (you chose: ${Q3.7/ChoiceGroup/SelectedChoices}), please use the "other" text box to specify the purpose for each of these special dietary plans. 


(Tick all options that are important in describing what you do)

- Religious/cultural reasons (1)
- Ethical/moral reasons (2)
- To achieve better quality training overall (3)
- To enhance muscle growth or recovery from training generally (4)
- To lose weight/body composition goals (5)
- Better health (6)
- Diagnosed allergies or intolerances (7)
- I enjoy aspects of this diet (e.g. the foods, variety, or flexibility involved) (10)
- An information source (e.g. someone told you or you read/watched it somewhere) told me to follow this diet (9)
- Other: Please specify (8) ________________________________________________

Display This Question:

If What is the main purpose of following a specific diet during ${q://QID26/ChoiceTextEntryValue/7}?... = An information source (e.g. someone told you or you read/watched it somewhere) told me to follow this diet

Q3.19 What information source told you to follow this diet for the time period specified (i.e. ${Q3.1/ChoiceTextEntryValue/7})? **(Tick all options that are relevant to informing your practice)**

- Coach (1)
- Sport nutritionist (4)
- Dietician (5)
- Medical Doctor (6)
- Physiotherapist (7)
- Family Member (8)
- A friend (9)
- Scientist (10)
- Training partner (11)
- Personal trainer (13)
- I read/watched it somewhere (e.g. blog, journal article, or Youtube) (12)
- Other: Please state. (14) ________________________________________________

End of Block: Nutrition for Competitive Phases

Start of Block: Hard Training Day Nutrition

Q4.1
Do you intentionally and purposefully eat more food/calories on all harder training days (i.e. a high volume and/or high intensity session)?
 (Tick all options that are important to describing what you do)

- Yes, I eat more food/calories in general (1)
- Yes, I eat more carbohydrate rich foods (2)
- Yes, I eat more protein rich foods (3)
- Yes, I eat more fat-rich foods or fat sources (4)
- Yes, I eat more protein drinks (5)
- Yes, I eat more protein bars (6)
- Yes, I eat more energy/protein bars (7)
- Yes, I drink more calorie-containing energy drinks (8)
- ⊗No, I do not eat more food/calories on all hard training days (9)

Display This Question:

If Do you intentionally and purposefully eat more food/calories on all harder training days (i.e. a... = Yes, I eat more food/calories in general

Or Do you intentionally and purposefully eat more food/calories on all harder training days (i.e. a... = Yes, I eat more carbohydrate rich foods

Or Do you intentionally and purposefully eat more food/calories on all harder training days (i.e. a... = Yes, I eat more protein rich foods

Or Do you intentionally and purposefully eat more food/calories on all harder training days (i.e. a... = Yes, I eat more fat-rich foods or fat sources

Or Do you intentionally and purposefully eat more food/calories on all harder training days (i.e. a... = Yes, I eat more protein drinks

Or Do you intentionally and purposefully eat more food/calories on all harder training days (i.e. a... = Yes, I eat more protein bars

Or Do you intentionally and purposefully eat more food/calories on all harder training days (i.e. a... = Yes, I eat more energy/protein bars

Or Do you intentionally and purposefully eat more food/calories on all harder training days (i.e. a... = Yes, I drink more calorie-containing energy drinks

Q4.2 When do you eat more food/calories on harder training days?**(Tick all options that are important to describing what you do)**

- I eat more at all meals (1)
- I eat more snacks (2)
- I eat more at main meals (3)
- I eat more within 4 hours before training (4)
- I eat more during training (5)
- I eat more in the 1 hour after training (6)
- I eat more just before bed (7)

Display This Question:

If Do you intentionally and purposefully eat more food/calories on all harder training days (i.e. a... = Yes, I eat more food/calories in general

Or Do you intentionally and purposefully eat more food/calories on all harder training days (i.e. a... = Yes, I eat more carbohydrate rich foods

Or Do you intentionally and purposefully eat more food/calories on all harder training days (i.e. a... = Yes, I eat more protein rich foods

Or Do you intentionally and purposefully eat more food/calories on all harder training days (i.e. a... = Yes, I eat more fat-rich foods or fat sources

Or Do you intentionally and purposefully eat more food/calories on all harder training days (i.e. a... = Yes, I eat more protein drinks

Or Do you intentionally and purposefully eat more food/calories on all harder training days (i.e. a... = Yes, I eat more protein bars

Or Do you intentionally and purposefully eat more food/calories on all harder training days (i.e. a... = Yes, I eat more energy/protein bars

Or Do you intentionally and purposefully eat more food/calories on all harder training days (i.e. a... = Yes, I drink more calorie-containing energy drinks

Q4.3 Why do you consume more food/calories on harder training days?**(Tick all options that are important to describing what you do)**

- I feel hungrier (1)
- I need more calories/fuel because of the higher training load (2)
- I don’t want to lose weight (3)
- Other: Please explain. (4) ________________________________________________

Display This Question:

If Do you intentionally and purposefully eat more food/calories on all harder training days (i.e. a... = Yes, I eat more food/calories in general

Or Do you intentionally and purposefully eat more food/calories on all harder training days (i.e. a... = Yes, I eat more carbohydrate rich foods

Or Do you intentionally and purposefully eat more food/calories on all harder training days (i.e. a... = Yes, I eat more protein rich foods

Or Do you intentionally and purposefully eat more food/calories on all harder training days (i.e. a... = Yes, I eat more fat-rich foods or fat sources

Or Do you intentionally and purposefully eat more food/calories on all harder training days (i.e. a... = Yes, I eat more protein drinks

Or Do you intentionally and purposefully eat more food/calories on all harder training days (i.e. a... = Yes, I eat more protein bars

Or Do you intentionally and purposefully eat more food/calories on all harder training days (i.e. a... = Yes, I eat more energy/protein bars

Or Do you intentionally and purposefully eat more food/calories on all harder training days (i.e. a... = Yes, I drink more calorie-containing energy drinks

Q4.4 Is there a source of information (e.g. someone told you or you read/watched it somewhere) that informs your practice of eating more on harder training days? 
(Tick all options that are important to describing what you do)

- Coach (1)
- Sport nutritionist (2)
- Dietician (3)
- Medical Doctor (4)
- Physiotherapist (5)
- Family Member (6)
- A friend (7)
- Scientist (8)
- Training partner (9)
- Personal trainer (11)
- I read/watched it somewhere (e.g. blog, journal article, or Youtube etc.) (10)
- Other: Please state. (12) ________________________________________________
- ⊗No specific source of information (14)

Display This Question:

If Do you intentionally and purposefully eat more food/calories on all harder training days (i.e. a... = No, I do not eat more food/calories on all hard training days

Q4.5 Why do you not eat more food/calories on hard training days?**(Tick all options that are important to describing what you do)**

- I don’t feel hungrier, so I don’t think I need it (1)
- I don’t think I need more calories/fuel on harder training days (2)
- Hard training sessions make me lose my appetite, so it is hard to eat more (3)
- I am already eating to a full stomach so there is no room for more (4)
- My social and/or financial situation makes it hard for me to get access to more food (5)
- Eating less on hard training days is an effective method for losing weight or achieving goals related to body composition (6)
- Other: Please state. (7) ________________________________________________

Display This Question:

If Do you intentionally and purposefully eat more food/calories on all harder training days (i.e. a... = No, I do not eat more food/calories on all hard training days

Q4.6 Is there a source of information (e.g. someone told you or you read/watched it somewhere) that informs your practice of not eating more on harder training days? **(Tick all options that are relevant to informing your practice)**

- Coach (1)
- Sport nutritionist (2)
- Dietician (3)
- Medical Doctor (4)
- Physiotherapist (5)
- Family Member (6)
- A friend (7)
- Scientist (8)
- Training partner (9)
- Personal trainer (11)
- I read/watched it somewhere (e.g. blog, journal article or Youtube etc.) (10)
- Other: Please state. (12) ________________________________________________
- ⊗No specific source of information (13)

End of Block: Hard Training Day Nutrition

Start of Block: Rest/Easy Training Day Nutrition

Q5.1
Do you intentionally and purposefully eat less food/calories on rest/easier training days (i.e. passive or active recovery or lower volume accessory days where none of the 3 powerlifting lifts are completed)? 
(Tick all options that are important to describing what you do)

- Yes, I eat less food/calories in general (1)
- Yes, I eat less carbohydrate rich foods (2)
- Yes, I eat less protein-rich foods (3)
- Yes, I eat less fat-rich foods or fat sources (4)
- Yes, I eat less protein drinks (5)
- Yes, I eat less protein bars (6)
- Yes, I eat less energy/protein bars (7)
- Yes, I drink less calorie-containing energy drinks (8)
- Yes, I eat more vegetables, fruits, or other low-energy foods/drinks to make up for reduced amount of other foods (9)
- ⊗No, I do not eat less food/calories on rest/easier training days (10)

Display This Question:

If Do you intentionally and purposefully eat less food/calories on rest/easier training days (i.e. p... = Yes, I eat less food/calories in general

Or Do you intentionally and purposefully eat less food/calories on rest/easier training days (i.e. p... = Yes, I eat less carbohydrate rich foods

Or Do you intentionally and purposefully eat less food/calories on rest/easier training days (i.e. p... = Yes, I eat less protein-rich foods

Or Do you intentionally and purposefully eat less food/calories on rest/easier training days (i.e. p... = Yes, I eat less fat-rich foods or fat sources

Or Do you intentionally and purposefully eat less food/calories on rest/easier training days (i.e. p... = Yes, I eat less protein drinks

Or Do you intentionally and purposefully eat less food/calories on rest/easier training days (i.e. p... = Yes, I eat less protein bars

Or Do you intentionally and purposefully eat less food/calories on rest/easier training days (i.e. p... = Yes, I eat less energy/protein bars

Or Do you intentionally and purposefully eat less food/calories on rest/easier training days (i.e. p... = Yes, I drink less calorie-containing energy drinks

Or Do you intentionally and purposefully eat less food/calories on rest/easier training days (i.e. p... = Yes, I eat more vegetables, fruits, or other low-energy foods/drinks to make up for reduced amount of other foods

Q5.2 When do you eat less food/calories on rest/easier training days? **(Tick all options that are important to describing what you do)**

- I eat less at all meals (1)
- I eat less snacks (2)
- I eat less at main meals (3)
- I eat less within 4 hours before training (4)
- I eat less during training (5)
- I eat less in the hour after training (6)
- I eat less just before bed (7)

Display This Question:

If Do you intentionally and purposefully eat less food/calories on rest/easier training days (i.e. p... = Yes, I eat less food/calories in general

Or Do you intentionally and purposefully eat less food/calories on rest/easier training days (i.e. p... = Yes, I eat less carbohydrate rich foods

Or Do you intentionally and purposefully eat less food/calories on rest/easier training days (i.e. p... = Yes, I eat less protein-rich foods

Or Do you intentionally and purposefully eat less food/calories on rest/easier training days (i.e. p... = Yes, I eat less fat-rich foods or fat sources

Or Do you intentionally and purposefully eat less food/calories on rest/easier training days (i.e. p... = Yes, I eat less protein drinks

Or Do you intentionally and purposefully eat less food/calories on rest/easier training days (i.e. p... = Yes, I eat less protein bars

Or Do you intentionally and purposefully eat less food/calories on rest/easier training days (i.e. p... = Yes, I eat less energy/protein bars

Or Do you intentionally and purposefully eat less food/calories on rest/easier training days (i.e. p... = Yes, I drink less calorie-containing energy drinks

Or Do you intentionally and purposefully eat less food/calories on rest/easier training days (i.e. p... = Yes, I eat more vegetables, fruits, or other low-energy foods/drinks to make up for reduced amount of other foods

Q5.3 Why do you consume less food/calories on rest/easier training days? **(Tick all options that are important to describing what you do)**

- I feel less hungry (1)
- I need fewer calories/fuel because I’m resting or have a low training load (2)
- This is an effective strategy to lose weight or achieve body composition related goals (3)
- Other: Please explain. (4) ________________________________________________

Display This Question:

If Do you intentionally and purposefully eat less food/calories on rest/easier training days (i.e. p... = Yes, I eat less food/calories in general

Or Do you intentionally and purposefully eat less food/calories on rest/easier training days (i.e. p... = Yes, I eat less carbohydrate rich foods

Or Do you intentionally and purposefully eat less food/calories on rest/easier training days (i.e. p... = Yes, I eat less protein-rich foods

Or Do you intentionally and purposefully eat less food/calories on rest/easier training days (i.e. p... = Yes, I eat less fat-rich foods or fat sources

Or Do you intentionally and purposefully eat less food/calories on rest/easier training days (i.e. p... = Yes, I eat less protein drinks

Or Do you intentionally and purposefully eat less food/calories on rest/easier training days (i.e. p... = Yes, I eat less protein bars

Or Do you intentionally and purposefully eat less food/calories on rest/easier training days (i.e. p... = Yes, I eat less energy/protein bars

Or Do you intentionally and purposefully eat less food/calories on rest/easier training days (i.e. p... = Yes, I drink less calorie-containing energy drinks

Or Do you intentionally and purposefully eat less food/calories on rest/easier training days (i.e. p... = Yes, I eat more vegetables, fruits, or other low-energy foods/drinks to make up for reduced amount of other foods

Q5.4 Is there a source of information (e.g. someone told you or you read/watched it somewhere) that informs your practice of eating less on rest/easy training days? **(Tick all options that are relevant to informing your practice)**

- Coach (1)
- Sport nutritionist (2)
- Dietician (3)
- Medical Doctor (4)
- Physiotherapist (5)
- Family Member (6)
- A friend (7)
- Scientist (8)
- Training partner (9)
- Personal trainer (11)
- I read/watched it somewhere (e.g. blog, journal article, or Youtube etc.) (10)
- Other: Please state. (12) ________________________________________________
- ⊗No specific source of information (13)

Display This Question:

If Do you intentionally and purposefully eat less food/calories on rest/easier training days (i.e. p... = No, I do not eat less food/calories on rest/easier training days

Q5.5 Why do you not eat less food/calories on rest/easier training days? **(Tick all options that are important to describing what you do)**

- I still feel hungry (1)
- I need to fuel up for future training days (2)
- I need calories for recovery and/or muscle growth (3)
- I don’t want to get ill or injured (4)
- I don’t want to lose weight (5)
- Other: Please state (6) ________________________________________________

Display This Question:

If Do you intentionally and purposefully eat less food/calories on rest/easier training days (i.e. p... = No, I do not eat less food/calories on rest/easier training days

Q5.6 Is there a source of information (e.g. someone told you or you read/watched it somewhere) that informs your practice of not eating less on rest/easy training days? (Tick all options that are relevant to informing your practice)

- Coach (1)
- Sport nutritionist (2)
- Dietician (3)
- Medical Doctor (4)
- Physiotherapist (5)
- Family Member (6)
- A friend (7)
- Scientist (8)
- Training partner (9)
- Personal trainer (11)
- I read/watched it somewhere (e.g. blog, journal article, or Youtube etc.) (10)
- Other: Please state. (12) ________________________________________________
- ⊗No specific source of information (13)

End of Block: Rest/Easy Training Day Nutrition

Start of Block: Pre-exercise Nutrition Practices for Key Sessions

Q6.1
Do you pay more attention to fuelling in the 1 to 4 hours before key training sessions?  

(Tick all options that are important in describing what you do)
 
**Note: A key training session is a high quality/intensity session consisting of at least one of the powerlifting movements (including variations/derivatives).**


**Note: Fuelling is eating foods (carbohydrate, protein, and fatty sources of foods, calorie containing drinks etc.) before training.**

- Yes, I eat more carbohydrate-rich foods before training (1)
- Yes, I eat more protein-rich foods before training (2)
- Yes, I eat more fat-rich foods and other fat sources (3)
- Yes, I eat more protein drinks (4)
- Yes, I eat more protein bars (5)
- Yes, I eat more energy/protein bars (6)
- Yes, I drink more calorie-containing energy drinks (7)
- Yes, I eat more foods in general before training (8)
- Yes, I focus on the optimal timing of my pre-training meal (9)
- ⊗No, I do not pay specific attention to fuelling in the 1 to 4 hours before key training sessions. (10)

Display This Question:

If Do you pay more attention to fuelling in the 1 to 4 hours before key training sessions?   (Tick a... = Yes, I eat more carbohydrate-rich foods before training

Or Do you pay more attention to fuelling in the 1 to 4 hours before key training sessions?   (Tick a... = Yes, I eat more protein-rich foods before training

Or Do you pay more attention to fuelling in the 1 to 4 hours before key training sessions?   (Tick a... = Yes, I eat more fat-rich foods and other fat sources

Or Do you pay more attention to fuelling in the 1 to 4 hours before key training sessions?   (Tick a... = Yes, I eat more protein drinks

Or Do you pay more attention to fuelling in the 1 to 4 hours before key training sessions?   (Tick a... = Yes, I eat more protein bars

Or Do you pay more attention to fuelling in the 1 to 4 hours before key training sessions?   (Tick a... = Yes, I eat more energy/protein bars

Or Do you pay more attention to fuelling in the 1 to 4 hours before key training sessions?   (Tick a... = Yes, I drink more calorie-containing energy drinks

Or Do you pay more attention to fuelling in the 1 to 4 hours before key training sessions?   (Tick a... = Yes, I eat more foods in general before training

Or Do you pay more attention to fuelling in the 1 to 4 hours before key training sessions?   (Tick a... = Yes, I focus on the optimal timing of my pre-training meal

Q6.2 Why do you pay attention to fuelling before key sessions? **(Tick all options that are important to describing what you do)**

- It will help me to train better during the upcoming session (1)
- I feel more energized for the whole day when I focus on fuelling before key sessions (2)
- It will reduce the risk of illness or injury (3)
- I don’t want to lose weight (4)
- I like to not feel hungry and/or have a full stomach going into a training session (5)
- Other: Please explain. (6) ________________________________________________

Display This Question:

If Do you pay more attention to fuelling in the 1 to 4 hours before key training sessions?   (Tick a... = Yes, I eat more carbohydrate-rich foods before training

Or Do you pay more attention to fuelling in the 1 to 4 hours before key training sessions?   (Tick a... = Yes, I eat more protein-rich foods before training

Or Do you pay more attention to fuelling in the 1 to 4 hours before key training sessions?   (Tick a... = Yes, I eat more fat-rich foods and other fat sources

Or Do you pay more attention to fuelling in the 1 to 4 hours before key training sessions?   (Tick a... = Yes, I eat more protein drinks

Or Do you pay more attention to fuelling in the 1 to 4 hours before key training sessions?   (Tick a... = Yes, I eat more protein bars

Or Do you pay more attention to fuelling in the 1 to 4 hours before key training sessions?   (Tick a... = Yes, I eat more energy/protein bars

Or Do you pay more attention to fuelling in the 1 to 4 hours before key training sessions?   (Tick a... = Yes, I drink more calorie-containing energy drinks

Or Do you pay more attention to fuelling in the 1 to 4 hours before key training sessions?   (Tick a... = Yes, I eat more foods in general before training

Or Do you pay more attention to fuelling in the 1 to 4 hours before key training sessions?   (Tick a... = Yes, I focus on the optimal timing of my pre-training meal

Q6.3 Is there a source of information (e.g. someone told you or you read/watched it somewhere) that informs your practice of paying attention to fuelling before key training sessions? **(Tick all options that are relevant to informing your practice)**

- Coach (1)
- Sport nutritionist (2)
- Dietician (3)
- Medical Doctor (4)
- Physiotherapist (5)
- Family Member (6)
- A friend (7)
- Scientist (8)
- Training partner (9)
- Personal trainer (11)
- I read/watched it somewhere (e.g. blog, journal article, or Youtube etc.) (10)
- Other: Please state. (12) ________________________________________________
- ⊗No specific source of information (13)

Display This Question:

If Do you pay more attention to fuelling in the 1 to 4 hours before key training sessions?   (Tick a... = No, I do not pay specific attention to fuelling in the 1 to 4 hours before key training sessions.

Q6.4 Why do you not pay specific attention to fuelling in the 1 to 4 hours before key training sessions?  **(Tick all options that are important to describing what you do)**

- I don’t feel it’s necessary to achieve better performance outcomes for that session (1)
- I’d like to but eating food before higher intensity/quality sessions makes me feel sick (2)
- My living arrangements and/or financial situation makes it hard for me to get access to suitable options (3)
- My weight/body composition goals prevent me from eating extra foods (4)
- I haven’t received any advice or instruction to guide a decision on fuelling before exercise (5)
- My living situation makes it difficult to implement dietary changes (e.g. my parent or partner does most of the food preparation) (6)
- Other: Please state. (7) ________________________________________________

Display This Question:

If Do you pay more attention to fuelling in the 1 to 4 hours before key training sessions?   (Tick a... = No, I do not pay specific attention to fuelling in the 1 to 4 hours before key training sessions.

Q6.5 Is there a source of information (e.g. someone told you or you read/watched it somewhere) that informs your practice of not paying attention to fuelling before key training sessions? **(Tick all options that are relevant to informing your practice)**

- Coach (1)
- Sport nutritionist (2)
- Dietician (3)
- Medical Doctor (4)
- Physiotherapist (5)
- Family Member (6)
- A friend (7)
- Scientist (8)
- Training partner (9)
- Personal trainer (11)
- I read/watched it somewhere (e.g. blog, journal article, or Youtube etc.) (10)
- Other: Please state. (12) ________________________________________________
- ⊗No specific source of information (13)

End of Block: Pre-exercise Nutrition Practices for Key Sessions

Start of Block: Post-training Meal Habits

Q7.1 Do you pay more attention to your nutrition after key training sessions (within 3 hours after the end of the key training session)?**(Tick all options that are important to describing what you do)**Note: A key training session is a high quality/intensity session consisting of at least one of the powerlifting movements (including variations/derivatives).

- Yes, I eat more carbohydrate-rich foods after training. (1)
- Yes, I eat more protein-rich foods after training (2)
- Yes, I eat more foods in general after training (3)
- Yes, I eat more protein drinks (4)
- Yes, I eat more protein bars (5)
- Yes, I eat more energy/protein bars (6)
- Yes, I drink more calorie-containing energy drinks (7)
- Yes, I focus on timing my post-workout meal within 1hr after training (8)
- ⊗No, I do not give specific attention to post-training nutrition after key sessions (within 3 hours) (9)

Display This Question:

If Do you pay more attention to your nutrition after key training sessions (within 3 hours after the... = Yes, I eat more carbohydrate-rich foods after training.

Or Do you pay more attention to your nutrition after key training sessions (within 3 hours after the... = Yes, I eat more protein-rich foods after training

Or Do you pay more attention to your nutrition after key training sessions (within 3 hours after the... = Yes, I eat more foods in general after training

Or Do you pay more attention to your nutrition after key training sessions (within 3 hours after the... = Yes, I eat more protein drinks

Or Do you pay more attention to your nutrition after key training sessions (within 3 hours after the... = Yes, I eat more protein bars

Or Do you pay more attention to your nutrition after key training sessions (within 3 hours after the... = Yes, I eat more energy/protein bars

Or Do you pay more attention to your nutrition after key training sessions (within 3 hours after the... = Yes, I drink more calorie-containing energy drinks

Or Do you pay more attention to your nutrition after key training sessions (within 3 hours after the... = Yes, I focus on timing my post-workout meal within 1hr after training

Q7.2 Why do you pay more attention to post-training nutrition after key sessions?**(Tick all options that are important to describing what you do)**

- It will specifically enhance the benefits (e.g. strength or muscle gain) from the session I have just finished (1)
- I recover better for the whole day when I focus on recovery immediately after key sessions (2)
- It reduces the risk of illness and injury (3)
- It helps me build/retain muscle mass (4)
- I’m hungry and I feel like eating at this time (5)
- ⊗I don't know (7)
- Other: Please state. (6) ________________________________________________

Display This Question:

If Do you pay more attention to your nutrition after key training sessions (within 3 hours after the... = Yes, I eat more carbohydrate-rich foods after training.

Or Do you pay more attention to your nutrition after key training sessions (within 3 hours after the... = Yes, I eat more protein-rich foods after training

Or Do you pay more attention to your nutrition after key training sessions (within 3 hours after the... = Yes, I eat more foods in general after training

Or Do you pay more attention to your nutrition after key training sessions (within 3 hours after the... = Yes, I eat more protein drinks

Or Do you pay more attention to your nutrition after key training sessions (within 3 hours after the... = Yes, I eat more protein bars

Or Do you pay more attention to your nutrition after key training sessions (within 3 hours after the... = Yes, I eat more energy/protein bars

Or Do you pay more attention to your nutrition after key training sessions (within 3 hours after the... = Yes, I drink more calorie-containing energy drinks

Or Do you pay more attention to your nutrition after key training sessions (within 3 hours after the... = Yes, I focus on timing my post-workout meal within 1hr after training

Q7.3 Is there a source of information (e.g. someone told you or you read/watched it somewhere) that informs your practice of paying attention to nutrition after key training sessions? **(Tick all options that are relevant to informing your practice)**

- Coach (1)
- Sport nutritionist (2)
- Dietician (3)
- Medical Doctor (4)
- Physiotherapist (5)
- Family Member (6)
- A friend (7)
- Scientist (8)
- Training partner (9)
- Personal trainer (11)
- I read/watched it somewhere (e.g. blog, journal article, or Youtube etc.) (10)
- Other: Please state. (12) ________________________________________________
- ⊗No specific source of information (13)

Display This Question:

If Do you pay more attention to your nutrition after key training sessions (within 3 hours after the... = No, I do not give specific attention to post-training nutrition after key sessions (within 3 hours)

Q7.4 Why do you not give specific attention to post-training nutrition after key sessions?**(Tick all options that are important to describing what you do)**

- It’s not necessary for that session (1)
- I’d like to, but I have no appetite after these sessions and/or eating food at this time makes me feel sick (2)
- My finances and/or living arrangements make it difficult to access suitable options (3)
- My weight/body composition concerns prevent me from eating extra foods (4)
- I haven’t received any special advice about post-training nutrition (5)
- My living situation makes it difficult to implement dietary changes (e.g. my parent or partner does most of the food preparation) (6)
- Other: Please explain (7) ________________________________________________

Display This Question:

If Do you pay more attention to your nutrition after key training sessions (within 3 hours after the... = No, I do not give specific attention to post-training nutrition after key sessions (within 3 hours)

Q7.5 Is there a source of information (e.g. someone told you or you read/watched it somewhere) that informs your practice of not paying attention to nutrition after key training sessions? **(Tick all options that are relevant to informing your practice)**

- Coach (1)
- Sport nutritionist (2)
- Dietician (3)
- Medical Doctor (4)
- Physiotherapist (5)
- Family Member (6)
- A friend (7)
- Scientist (8)
- Training partner (9)
- Personal trainer (11)
- I read/watched it somewhere (e.g. blog, journal article, or Youtube etc.) (10)
- Other: Please state. (12) ________________________________________________
- ⊗No specific source of information (13)

End of Block: Post-training Meal Habits

Start of Block: Fasted Training

Q8.1 Do you intentionally complete training sessions in the fasted state?**(Tick all options that are important to describing what you do)**Note: Fasted training is the completion of a training session without eating food or calorie containing drinks in the 8 hours prior (e.g. training after an overnight fast or an afternoon training session without eating for 8 hours prior).

- Yes, I complete all my key training sessions in the fasted state (1)
- Yes, I complete some key training sessions in the fasted state (2)
- Yes, I complete all my easier training sessions in the fasted state (3)
- Yes, I complete some of my easier training sessions in the fasted state (4)
- ⊗No, I do not train in the fasted state (5)

Display This Question:

If Do you intentionally complete training sessions in the fasted state?  (Tick all options that are... = Yes, I complete all my key training sessions in the fasted state

Or Do you intentionally complete training sessions in the fasted state?  (Tick all options that are... = Yes, I complete some key training sessions in the fasted state

Or Do you intentionally complete training sessions in the fasted state?  (Tick all options that are... = Yes, I complete all my easier training sessions in the fasted state

Or Do you intentionally complete training sessions in the fasted state?  (Tick all options that are... = Yes, I complete some of my easier training sessions in the fasted state

Q8.2 When do you train in the fasted state?**(Tick all options that are important to describing what you do)**

- In the morning after an overnight fast (1)
- In the afternoon after at least 8 hours of fasting (2)
- Other: Please explain (3) ________________________________________________

Display This Question:

If Do you intentionally complete training sessions in the fasted state?  (Tick all options that are... = Yes, I complete all my key training sessions in the fasted state

Or Do you intentionally complete training sessions in the fasted state?  (Tick all options that are... = Yes, I complete some key training sessions in the fasted state

Or Do you intentionally complete training sessions in the fasted state?  (Tick all options that are... = Yes, I complete all my easier training sessions in the fasted state

Or Do you intentionally complete training sessions in the fasted state?  (Tick all options that are... = Yes, I complete some of my easier training sessions in the fasted state

Q8.3 How often do you train in the fasted state?

- Once a week (1)
- Twice a week (2)
- Three times a week (3)
- More than three times per week (4)

Display This Question:

If Do you intentionally complete training sessions in the fasted state?  (Tick all options that are... = Yes, I complete all my key training sessions in the fasted state

Or Do you intentionally complete training sessions in the fasted state?  (Tick all options that are... = Yes, I complete some key training sessions in the fasted state

Or Do you intentionally complete training sessions in the fasted state?  (Tick all options that are... = Yes, I complete all my easier training sessions in the fasted state

Or Do you intentionally complete training sessions in the fasted state?  (Tick all options that are... = Yes, I complete some of my easier training sessions in the fasted state

Q8.4 Why do you train in the fasted state?**(Tick all options that are important to describing what you do)**

- I find it helps my overall training (1)
- I like to train fasted because it helps with stomach comfort (2)
- It’s more convenient to go straight to training without eating (3)
- It helps with weight loss and/or body composition goals (4)
- Other: Please state (5) ________________________________________________

Display This Question:

If Do you intentionally complete training sessions in the fasted state?  (Tick all options that are... = Yes, I complete all my key training sessions in the fasted state

Or Do you intentionally complete training sessions in the fasted state?  (Tick all options that are... = Yes, I complete some key training sessions in the fasted state

Or Do you intentionally complete training sessions in the fasted state?  (Tick all options that are... = Yes, I complete all my easier training sessions in the fasted state

Or Do you intentionally complete training sessions in the fasted state?  (Tick all options that are... = Yes, I complete some of my easier training sessions in the fasted state

Q8.5 Is there a source of information (e.g. someone told you or you read/watched it somewhere) that informs your practice of training in the fasted state? **(Tick all options that are relevant to informing your practice)**

- Coach (1)
- Sport nutritionist (2)
- Dietician (3)
- Medical Doctor (4)
- Physiotherapist (5)
- Family Member (6)
- A friend (7)
- Scientist (8)
- Training partner (9)
- Personal trainer (11)
- I read/watched it somewhere (e.g. blog, journal article, or Youtube etc.) (10)
- Other: Please state. (12) ________________________________________________
- ⊗No specific source of information (13)

Display This Question:

If Do you intentionally complete training sessions in the fasted state?  (Tick all options that are... = No, I do not train in the fasted state

Q8.6 Why do you not perform training sessions in the fasted state? **(Tick all options that are important to describing what you do)**

- I do not like to train on an empty stomach/when I’m hungry (1)
- It impairs my performance during that specific training session (2)
- I don’t go 8 hours without eating (3)
- Other: Please explain (4) ________________________________________________

Display This Question:

If Do you intentionally complete training sessions in the fasted state?  (Tick all options that are... = No, I do not train in the fasted state

Q8.7 Is there a source of information (e.g. someone told you or you read/watched it somewhere) that informs your practice of not training in the fasted state? **(Tick all options that are relevant to informing your practice)**

- Coach (1)
- Sport nutritionist (2)
- Dietician (3)
- Medical Doctor (4)
- Physiotherapist (5)
- Family Member (6)
- A friend (7)
- Scientist (8)
- Training partner (9)
- Personal trainer (11)
- I read/watched it somewhere (e.g. blog, journal article, or Youtube etc.) (10)
- Other: Please state. (12) ________________________________________________
- ⊗No specific source of information (13)

End of Block: Fasted Training

Start of Block: Intra-session Nutrition

Q9.1 Do you consume food and/or calorie containing drinks during training sessions? **(Tick all options that are important to describing what you do)**   Note: A key training session is a high quality/intensity session consisting of at least one of the powerlifting movements (including variations/derivatives).
 Note: Easier training sessions are rest or lower volume/intensity days (e.g. passive or active recovery days or lower volume accessory days) where none of the 3 powerlifting lifts are completed.

- ⊗Yes, for all training sessions, easy or hard (4)
- ⊗Yes, for all key training sessions (1)
- ⊗Yes, for all key sessions and occasionally for easy sessions (7)
- ⊗Yes, occasionally for key training sessions (2)
- ⊗Yes, occasionally for a mix of easy and hard training sessions (3)
- ⊗Yes, other: Please specify (5) ________________________________________________
- ⊗No, I do not consume food and/or calorie containing drinks during training sessions (6)

Display This Question:

If Do you consume food and/or calorie containing drinks during training sessions?  (Tick all options... = Yes, for all key training sessions

Or Do you consume food and/or calorie containing drinks during training sessions?  (Tick all options... = Yes, occasionally for key training sessions

Or Do you consume food and/or calorie containing drinks during training sessions?  (Tick all options... = Yes, occasionally for a mix of easy and hard training sessions

Or Do you consume food and/or calorie containing drinks during training sessions?  (Tick all options... = Yes, for all training sessions, easy or hard

Or Do you consume food and/or calorie containing drinks during training sessions?  (Tick all options... = Yes, other: Please specify

Or Do you consume food and/or calorie containing drinks during training sessions?  (Tick all options... = Yes, for all key sessions and occasionally for easy sessions

Q9.2
What kind of foods and/or calorie containing drinks do you consume during a training session? 
(Tick all options that are important to describing what you do)

- I eat more carbohydrate-rich foods during training (e.g. sugary sweets/lollies, fruits, grains) (1)
- I eat more protein-rich foods during training (e.g. eggs, meats, whey protein) (2)
- I eat more foods in general during training (3)
- I eat more protein drinks (4)
- I eat more protein bars (5)
- I eat more energy/protein bars (6)
- I drink more calorie-containing energy drinks (7)
- Other: Please specify. (8) ________________________________________________

Display This Question:

If Do you consume food and/or calorie containing drinks during training sessions?  (Tick all options... = Yes, for all key training sessions

Or Do you consume food and/or calorie containing drinks during training sessions?  (Tick all options... = Yes, occasionally for key training sessions

Or Do you consume food and/or calorie containing drinks during training sessions?  (Tick all options... = Yes, occasionally for a mix of easy and hard training sessions

Or Do you consume food and/or calorie containing drinks during training sessions?  (Tick all options... = Yes, for all training sessions, easy or hard

Or Do you consume food and/or calorie containing drinks during training sessions?  (Tick all options... = Yes, other: Please specify

Or Do you consume food and/or calorie containing drinks during training sessions?  (Tick all options... = Yes, for all key sessions and occasionally for easy sessions

Q9.3 Why do you consume food/drink during the training session?**(Tick all options that are important to describing what you do)**

- I find it helps me train better at that specific session (1)
- I can’t actually tell, but I believe it is supposed to make me train better (2)
- It’s a part of consuming enough calories over the day when I am training hard (3)
- It makes me feel less hungry and/or boosts energy levels (6)
- ⊗I don’t know (4)
- Other: Please explain (5) ________________________________________________

Display This Question:

If Do you consume food and/or calorie containing drinks during training sessions?  (Tick all options... = Yes, for all key training sessions

Or Do you consume food and/or calorie containing drinks during training sessions?  (Tick all options... = Yes, occasionally for key training sessions

Or Do you consume food and/or calorie containing drinks during training sessions?  (Tick all options... = Yes, occasionally for a mix of easy and hard training sessions

Or Do you consume food and/or calorie containing drinks during training sessions?  (Tick all options... = Yes, for all training sessions, easy or hard

Or Do you consume food and/or calorie containing drinks during training sessions?  (Tick all options... = Yes, other: Please specify

Or Do you consume food and/or calorie containing drinks during training sessions?  (Tick all options... = Yes, for all key sessions and occasionally for easy sessions

Q9.4 Is there a source of information (e.g. someone told you or you read/watched it somewhere) that informs your practice of consuming foods and/or calorie containing drinks during training sessions? **(Tick all options that are relevant to informing your practice)**

- Coach (1)
- Sport nutritionist (2)
- Dietician (3)
- Medical Doctor (4)
- Physiotherapist (5)
- Family Member (6)
- A friend (7)
- Scientist (8)
- Training partner (9)
- Personal trainer (11)
- I read/watched it somewhere (e.g. blog, journal article, or Youtube etc.) (10)
- Other: Please state. (12) ________________________________________________
- ⊗No specific source of information (13)

Display This Question:

If Do you consume food and/or calorie containing drinks during training sessions?  (Tick all options... = No, I do not consume food and/or calorie containing drinks during training sessions

Q9.5 Why do you not consume foods and/or calorie containing drinks during training sessions?**(Tick all options that are important to describing what you do)**

- I don’t think it helps my training in any way (1)
- My stomach gets upset when eating/drinking during a training session (2)
- I can’t fit extra energy intake into my overall dietary plan (4)
- I had not considered this nutrition approach before (3)
- ⊗I don't know (6)
- Other: Please state (5) ________________________________________________

Display This Question:

If Do you consume food and/or calorie containing drinks during training sessions?  (Tick all options... = No, I do not consume food and/or calorie containing drinks during training sessions

Q9.6 Is there a source of information (e.g. someone told you or you read/watched it somewhere) that informs your practice of not consuming foods and/or calorie containing drinks during training sessions? **(Tick all options that are relevant to informing your practice)**

- Coach (1)
- Sport nutritionist (2)
- Dietician (3)
- Medical Doctor (4)
- Physiotherapist (5)
- Family Member (6)
- A friend (7)
- Scientist (8)
- Training partner (9)
- Personal trainer (11)
- I read/watched it somewhere (e.g. blog, journal article, or Youtube etc.) (10)
- Other: Please state. (12) ________________________________________________
- ⊗No specific source of information (13)

End of Block: Intra-session Nutrition

Start of Block: Pre-training Supplementation Practices

Q10.1
Do you pay attention to supplementation (e.g. a pre-workout formula, caffeine, creatine, glutamine etc.) in the 2 hours preceding training sessions?

(Tick the option most important to describing what you do)
   Note: A key training session is a high quality/intensity session consisting of at least one of the powerlifting movements (including variations/derivatives).   Note: Easier training sessions are rest or lower volume/intensity days (e.g. passive or active recovery days or lower volume accessory days) where none of the 3 powerlifting lifts are completed.

- ⊗Yes, for all training sessions, easy or hard (4)
- ⊗Yes, for all key training sessions (1)
- ⊗Yes, for all key sessions and occasionally for easy sessions (7)
- ⊗Yes, occasionally for key training sessions (2)
- ⊗Yes, occasionally for a mix of easy and hard training sessions (3)
- ⊗Yes, other: Please specify (5) ________________________________________________
- ⊗No, I do not pay attention to supplementation before training sessions (6)

Display This Question:

If Do you pay attention to supplementation (e.g. a pre-workout formula, caffeine, creatine, glutamin... = Yes, for all training sessions, easy or hard

Or Do you pay attention to supplementation (e.g. a pre-workout formula, caffeine, creatine, glutamin... = Yes, for all key training sessions

Or Do you pay attention to supplementation (e.g. a pre-workout formula, caffeine, creatine, glutamin... = Yes, for all key sessions and occasionally for easy sessions

Or Do you pay attention to supplementation (e.g. a pre-workout formula, caffeine, creatine, glutamin... = Yes, occasionally for key training sessions

Or Do you pay attention to supplementation (e.g. a pre-workout formula, caffeine, creatine, glutamin... = Yes, occasionally for a mix of easy and hard training sessions

Or Do you pay attention to supplementation (e.g. a pre-workout formula, caffeine, creatine, glutamin... = Yes, other: Please specify

Q107 Why do you use supplementation prior to the training session (within 2 hours)? 
(Tick all options that are important to describing what you do)
 
Note: A key training session is a high quality/intensity session consisting of at least one of the powerlifting movements (including variations/derivatives).
Note: Easier training sessions are rest or lower volume/intensity days (e.g. passive or active recovery days or lower volume accessory days) where none of the 3 powerlifting lifts are completed.

- It helps me perform better at that specific training session (1)
- It helps to make me feel fuller for the training session (4)
- It helps me be more awake or alert (5)
- ⊗I don’t know (6)
- Other: Please specify (7)

Display This Question:

If Do you pay attention to supplementation (e.g. a pre-workout formula, caffeine, creatine, glutamin... = Yes, for all key training sessions

Or Do you pay attention to supplementation (e.g. a pre-workout formula, caffeine, creatine, glutamin... = Yes, occasionally for key training sessions

Or Do you pay attention to supplementation (e.g. a pre-workout formula, caffeine, creatine, glutamin... = Yes, occasionally for a mix of easy and hard training sessions

Or Do you pay attention to supplementation (e.g. a pre-workout formula, caffeine, creatine, glutamin... = Yes, for all training sessions, easy or hard

Or Do you pay attention to supplementation (e.g. a pre-workout formula, caffeine, creatine, glutamin... = Yes, other: Please specify

Or Do you pay attention to supplementation (e.g. a pre-workout formula, caffeine, creatine, glutamin... = Yes, for all key sessions and occasionally for easy sessions

Q10.7
What supplements do you use before a training session (within 2 hours)? 
(Tick all options that are important to describing what you do)

- A pre-workout formula (any combination of caffeine, creatine, vitamins, minerals, amino acids, lactate buffers). (1)
- Caffeine pills (2)
- Energy drink (3)
- Protein shake (4)
- Branch Chain Amino Acids (BCAA’s) (5)
- Beta-alanine (6)
- Glutamine (7)
- Creatine (8)
- Nitric oxide precursors (9)
- Other: Please state, typed answer. (10) ________________________________________________

Display This Question:

If Do you pay attention to supplementation (e.g. a pre-workout formula, caffeine, creatine, glutamin... = Yes, for all key training sessions

Or Do you pay attention to supplementation (e.g. a pre-workout formula, caffeine, creatine, glutamin... = Yes, occasionally for key training sessions

Or Do you pay attention to supplementation (e.g. a pre-workout formula, caffeine, creatine, glutamin... = Yes, occasionally for a mix of easy and hard training sessions

Or Do you pay attention to supplementation (e.g. a pre-workout formula, caffeine, creatine, glutamin... = Yes, for all training sessions, easy or hard

Or Do you pay attention to supplementation (e.g. a pre-workout formula, caffeine, creatine, glutamin... = Yes, other: Please specify

Or Do you pay attention to supplementation (e.g. a pre-workout formula, caffeine, creatine, glutamin... = Yes, for all key sessions and occasionally for easy sessions

Q10.8 Is there a source of information (e.g. someone told you or you read/watched it somewhere) that informs your practice of paying attention to supplementation before training sessions? **(Tick all options that are relevant to informing your practice)**

- Coach (1)
- Sport nutritionist (2)
- Dietician (3)
- Medical Doctor (4)
- Physiotherapist (5)
- Family Member (6)
- A friend (7)
- Scientist (8)
- Training partner (9)
- Personal trainer (11)
- I read/watched it somewhere (e.g. blog, journal article, or Youtube etc.) (10)
- Other: Please state. (12) ________________________________________________
- ⊗No specific source of information (13)

Display This Question:

If Do you pay attention to supplementation (e.g. a pre-workout formula, caffeine, creatine, glutamin... = No, I do not pay attention to supplementation before training sessions

Q10.9
Why do you not use supplementation prior to training sessions (within 2 hours)?
 (Tick all options that are important to describing what you do)

- My lifestyle and/or financial situation makes it tough to do so (1)
- I don’t think they are necessary for me to perform well (2)
- They cause my stomach to become upset (3)
- I experience adverse side effects (e.g. dizziness, rapid heart rate, intensified nervousness and/or anxiety) (4)
- ⊗I don't know (6)
- Other: Please state (5) ________________________________________________

Display This Question:

If Do you pay attention to supplementation (e.g. a pre-workout formula, caffeine, creatine, glutamin... = No, I do not pay attention to supplementation before training sessions

Q10.10 Is there a source of information (e.g. someone told you or you read/watched it somewhere) that informs your practice of not paying attention to supplementation before training sessions? **(Tick all options that are relevant to informing your practice)**

- Coach (1)
- Sport nutritionist (2)
- Dietician (3)
- Medical Doctor (4)
- Physiotherapist (5)
- Family Member (6)
- A friend (7)
- Scientist (8)
- Training partner (9)
- Personal trainer (11)
- I read/watched it somewhere (e.g. blog, journal article, or Youtube etc.) (10)
- Other: Please state. (12) ________________________________________________
- ⊗No specific source of information (13)

End of Block: Pre-training Supplementation Practices
