# Supplementary material for: The general nutrition practices of competitive powerlifters vary by competitive calibre and sex, weight, and age class
Source: Eur J Nutr. 2023 Aug 16;62(8):3297–310. doi: 10.1007/s00394-023-03233-6 (PMC10611852; doi:10.1007/s00394-023-03233-6)
Supplement: Supplementary file 2 — Supplementary file2 (DOCX 19 KB) [file 394_2023_3233_MOESM2_ESM.docx]

**Title**

The General Nutrition Practices of Competitive Powerlifters Vary by Competitive Calibre and Sex, Weight, and Age Class

**Authors**

Andrew King^1^, Kedric Kwan^1^, Ivan Jukic^1,2^, Caryn Zinn^1^, and Eric Helms^1^

**Affiliations**

^1^Sport Performance Research Institute New Zealand (SPRINZ), Auckland University of Technology, Auckland, New Zealand

^2^School of Engineering, Computer and Mathematical Sciences, Auckland University of Technology, Auckland, New Zealand

**ORCID**

Andrew King: https://orcid.org/0000-0002-9332-6506

Ivan Jukic: https://orcid.org/0000-0002-0900-9410

**Corresponding Author**

Andrew King

PhD Candidate

Sport Performance Research Institute New Zealand (SPRINZ)

Auckland University of Technology

17 Antares Place, Mairangi Bay

Auckland, New Zealand, 0632

Email: andrew.king@aut.ac.nz

**Supplementary File II:**

**Competition Preparation: Reasons for following a specific diet.**

**Dietary Introduction for the Transition Phase**

Do you deliberately but periodically introduce a special dietary plan to support your training goals during the ***transition*** phase?

Yes (n = 26)

What special dietary plan do you follow for the ***transition*** phase?

If It Fits Your Macros (IIFYM)/flexible dieting (n = 19)

High carb (n = 5)

Very high energy/extra energy (n = 5)

High protein, low carb (n = 2)

High protein, high carb (n = 2)

Reverse Dieting (n = 1)

Vegan/Vegetarian (n = 1)

Restricted energy (n = 1)

What is the main purpose of following an IIFYM/flexible diet during the ***transition*** phase?

Ethical/moral reasons (n = 1)

To achieve better quality training (n = 10)

To enhance muscle growth/recovery (n = 11)

To lose weight/body composition goals (n = 7)

Better health (n = 3)

I enjoy aspects of this diet (n = 7)

An information source told me to (n = 1, a scientist)

What is the main purpose of following a high carb diet during the ***transition*** phase?

To achieve better quality training (n = 4)

To enhance muscle growth/recovery (n = 4)

Better health (n = 1)

I enjoy aspects of this diet (n = 1)

What is the main purpose of following a very high energy/extra energy diet during the ***transition*** phase?

To achieve better quality training (n = 5)

To enhance muscle growth/recovery (n = 5)

Better health (n = 1)

An information source told me to (n = 1, I read/watched it somewhere)

What is the main purpose of following a high protein, low carb diet during the ***transition*** phase?

To achieve better quality training (n = 2)

To enhance muscle growth/recovery (n = 1)

Better health (n = 1)

What is the main purpose of following a high protein, high carb diet during the ***transition*** phase?

To achieve better quality training (n = 1)

To enhance muscle growth/recovery (n = 2)

I enjoy aspects of this diet (n = 1)

What is the main purpose of following a reverse diet during the ***transition*** phase?

To lose weight/body composition goals (n = 1)

An information source told me to (n = 1, dietician)

What is the main purpose of following a vegan/vegetarian diet during the ***transition*** phase?

Ethical/moral reasons (n = 1)

What is the main purpose of following a restricted energy diet during the ***transition*** phase?

To lose weight/body composition goals (n = 1)

Better health (n = 1)

There were no sub-group differences for any of the questions regarding nutrition practices during the transition phase (p > 0.05).

**Dietary Introduction for a Return from Injury**

Do you deliberately but periodically introduce a special dietary plan to support your training goals during the ***return from injury*** phase?

Yes (n = 16)

What special dietary plan do you follow for a return from injury?

IIFYM/flexible dieting (n = 8)

Very high energy/extra energy (n = 8)

High protein, low carb (n = 4)

High carb (n = 1)

Vegan/vegetarian (n = 1)

Paleo for athletes (n = 1)

LCHF (n = 1)

What is the main purpose of following an IIFYM/flexible diet during the ***transition*** phase?

To achieve better quality training (n = 4)

To enhance muscle growth/recovery (n = 6)

To lose weight/body composition goals (n = 2)

Better health (n = 6)

I enjoy aspects of this diet (n = 3)

An information source told me to (n = 1, sport nutritionist & dietician)

What is the main purpose of following a very high energy/extra energy diet during the ***transition*** phase?

To achieve better quality training (n = 6)

To enhance muscle growth/recovery (n = 7)

To lose weight/body composition goals (n = 1)

Better health (n = 6)

What is the main purpose of following a high protein, low carb diet during the ***transition*** phase?

To achieve better quality training (n = 2)

To enhance muscle growth/recovery (n = 2)

To lose weight/body composition goals (n = 1)

Better health (n = 2)

What is the main purpose of following a high carb diet during the ***transition*** phase?

To achieve better quality training (n = 1)

What is the main purpose of following a vegan/vegetarian diet during the ***transition*** phase?

Ethical/moral reasons (n = 1)

What is the main purpose of following a paleo for athletes’ diet during the ***transition*** phase?

To achieve better quality training (n = 1)

Better health (n = 1)

What is the main purpose of following a LCHF diet during the ***transition*** phase?

To lose weight/body composition goals (n = 1)

There were no sub-group differences for any of the questions regarding nutrition practices during a return from injury (p > 0.05).
